# Supplementary material for: Cytotoxic and proliferation-inhibitory activity of natural and synthetic fungal tropolone sesquiterpenoids in various cell lines
Source: Heliyon. 2024 Sep 13;10(22):e37713. doi: 10.1016/j.heliyon.2024.e37713 (PMC11693898; doi:10.1016/j.heliyon.2024.e37713)
Supplement: Multimedia component 1 [file mmc1.pdf]

Supplementary Information

Cytotoxic and proliferation-inhibitory activity of natural and synthetic fungal tropolone sesquiterpenoids in various cell lines

*Timna C. Bergmann[1], Max Menssen[2], Carsten Schotte[3], Russell J. Cox[3], Cornelia Lee-Thedieck[1]**

T.C. Bergmann, Prof. Dr. C. Lee-Thedieck

[1] Institute of Cell Biology and Biophysics, Department of Cell Biology, Leibniz University Hannover, 30419 Hannover, Germany

Dr. M. Menssen

[2] Institute of Cell Biology and Biophysics, Department of Biostatistics, Leibniz University Hannover, 30419 Hannover, Germany

Dr. C. Schotte, Prof. Dr. R.J.Cox

[3] Institute of Organic Chemistry and BMWZ, Leibniz University Hannover, 30167 Hannover, Germany

* Prof. Dr. Cornelia Lee-Thedieck, Leibniz University Hannover, Institute of Cell Biology and Biophysics, Herrenhäuserstr 2, 30419 Hannover. Phone: +49 511 762 14 972; Fax: +49 511 762 2606; Email: lee-thedieck@cell.uni-hannover.de

Content

1. Supplementary Figures…………………………………..3

2. Statistical Supplementary Information……….20

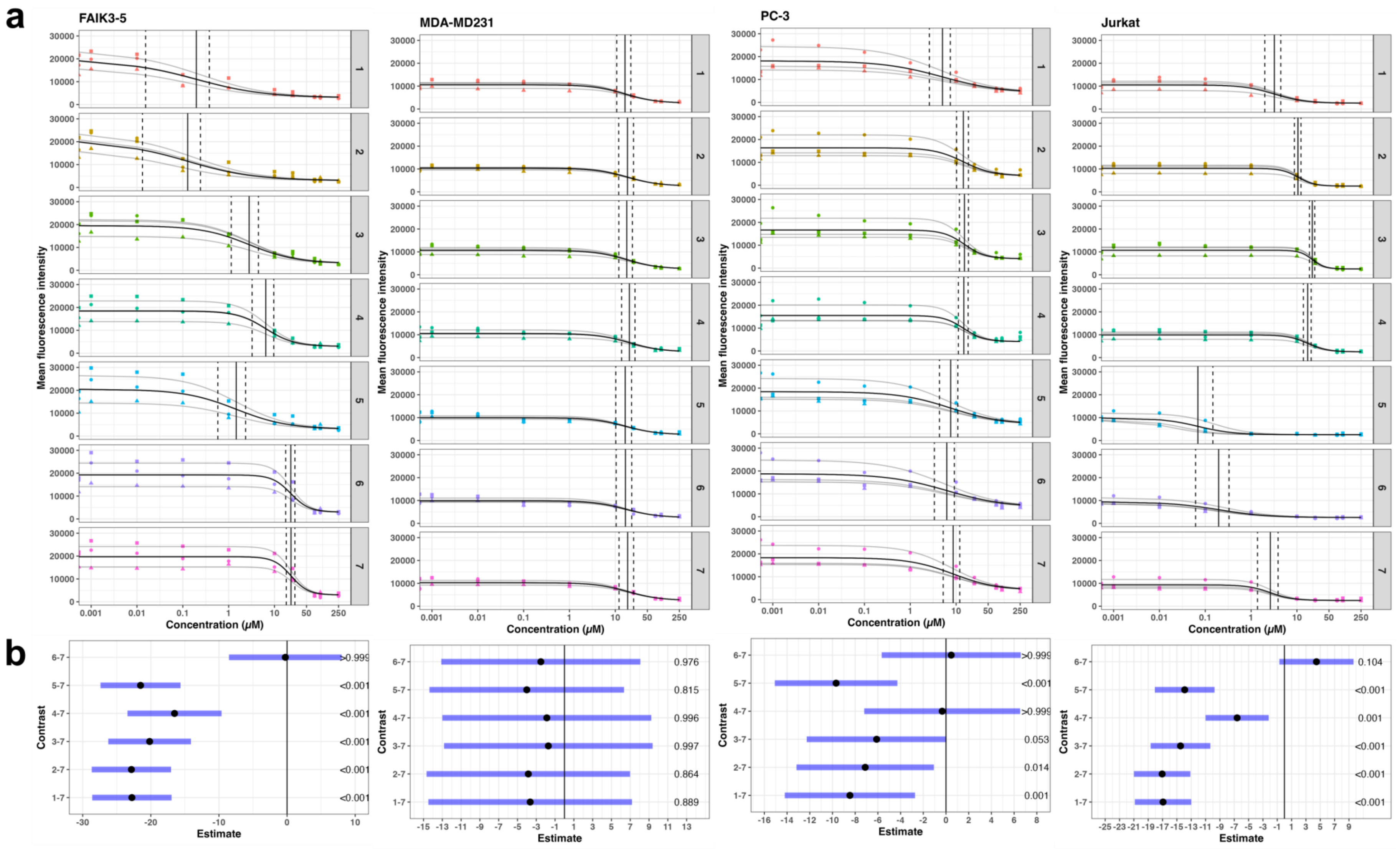

Figure S 1. (Legend next page)

Figure S 1. IC_{50} of murine FAIK3–5 cells and the cancer cell lines MDA–MB–231, PC–3 and Jurkat after treatment with 0.001 µM, 0.001 µM, 0.1 µM, 1 µM, 10 µM, 25 µM and 75 µM of the tropolone sesquiterpenoids (TS) pycnidione (**1**), eupenifeldin (**2**), xenovulene B (**3**), 4-hydroxyxenovulene B (**4**), 4-dehydroxy norpycnidione (**5**) and tropolone-lacking compound 4-hydroxy norxenovulene B (**6**) and 0.0001 % (v/v), 0.001 % (v/v), 0.01 % (v/v), 0.1 % (v/v), 1 % (v/v), 2.5 % (v/v) and 7.5 % (v/v) of their respective DMSO vehicle controls (7). **(a)** For each experimental repetition one according dose–response curve (grey) was modelled, resulting in the final mean dose-response curve (black) from which IC_{50} values (black vertical line) and the corresponding upper and lower confidence intervals (black dotted vertical lines) were derived. Mean fluorescence intensities were measured in three biological replicates (●=1, ▲=2, ■=3) deriving from the mean values of three technical replicates per experimental repetition. To accurately estimate the lower asymptote of each dose-response curve, dead controls without TS compounds were included comprising 10 % (v/v) and 25 % (v/v) DMSO. **(b)** Pairwise mean comparisons have been calculated to show statistically significant differences between calculated IC_{50} of DMSO vehicle controls and TS compound treated samples. Black dots indicate the estimated IC_{50} values and their 95 % confidence intervals (blue bars). The black vertical line indicate the Null hypothesis that the compared IC_{50} values differ from each other. The corresponding p-values for each calculation are displayed on the right side. Further details on modelling and IC_{50} derivation are given in the statistical supplementary statS1

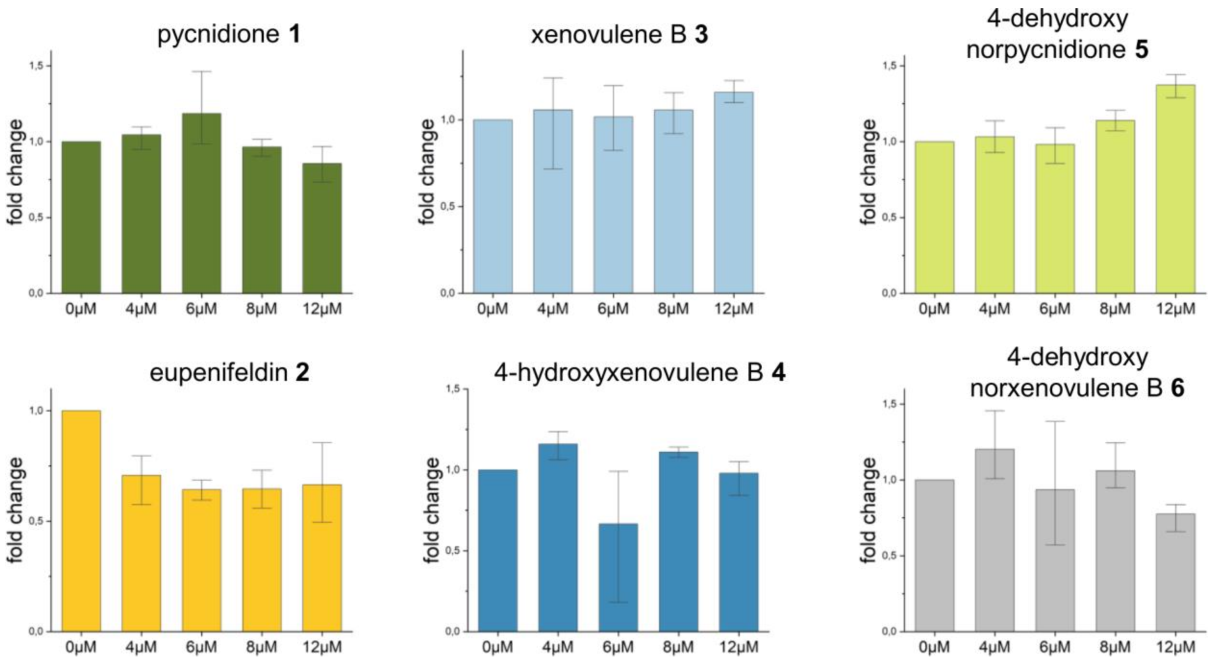

Figure S 2. Assessment of basic erythropoietin concentrations after administration of 4 µM, 6 µM, 8 µM and 12 µM of the tropolone sesquiterpenoids (TS) pycnidione **1**, eupenifeldin **2**, xenovulene B **3**, 4-hydroxyxenovulene B **4** and 4-dehydroxy norpycnidione **5** and the tropolone-moiety lacking compound 4-hydroxy norxenovulene B **6** for 24h (N=1 independent experiment). Initial tests on EPO concentrations were conducted to assess optimal compound concentration showing highest fold induction for pycnidione **1** at 6 µM and for 4 – dehydroxy norpycnidione **5** at 12 µM. Protein concentrations were obtained via ELISA and fold changes compared to the untreated control (0 µM) were calculated. Error bars indicate the standard deviation of the n = 3 technical replicates.

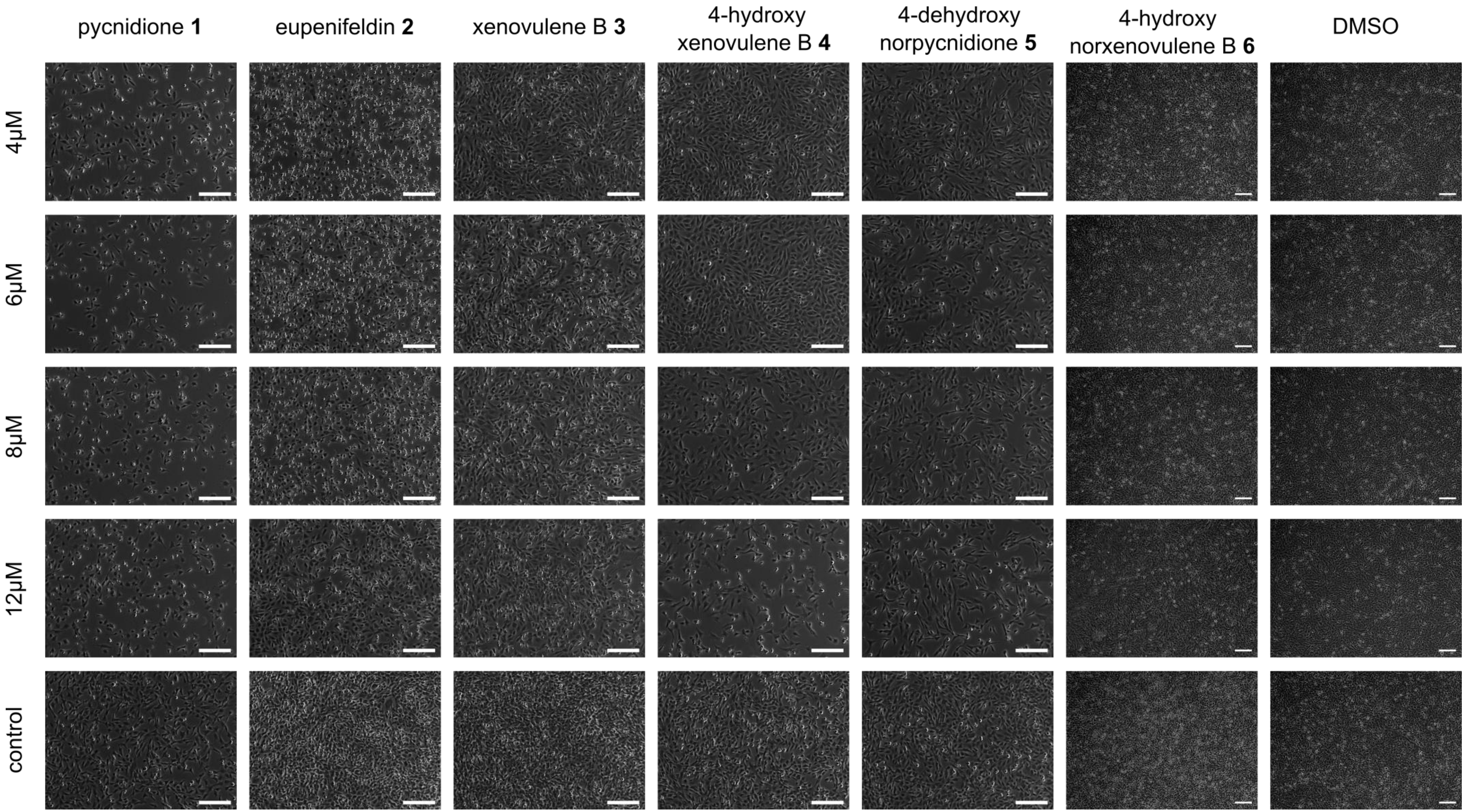

Figure S 3. (Legend next page)

Figure S 3. Phase contrast images of FAIK3-5 cell samples treated with 4 µM, 6 µM, 8 µM, and 12 µM of tropolone-sesquiterpenoids (TS) (pycnidione **1**, eupenifeldin **2**, xenovulene B **3**, 4-hydroxyxenovulene B **4** and 4-dehydroxy norpycnidione **5**), tropolone-lacking compound 4-hydoxy norxenovulene B **6**, the vehicle control dimethyl sulfoxide (DMSO) or cell culture medium (control) for 24 h. Scale bar = 100µm. TS treatment with compounds **1**, **2**, **3** and **5** induce a reduced cell density compared to the respective medium control, regardless of the compound concentration applied, whereas compound **4** shows a reduced cell confluence with higher concentrations of the compound. Treatment with tropolone-lacking compound **6** and DMSO does not influence cell densities in any applied concentration compared to the medium control.

a

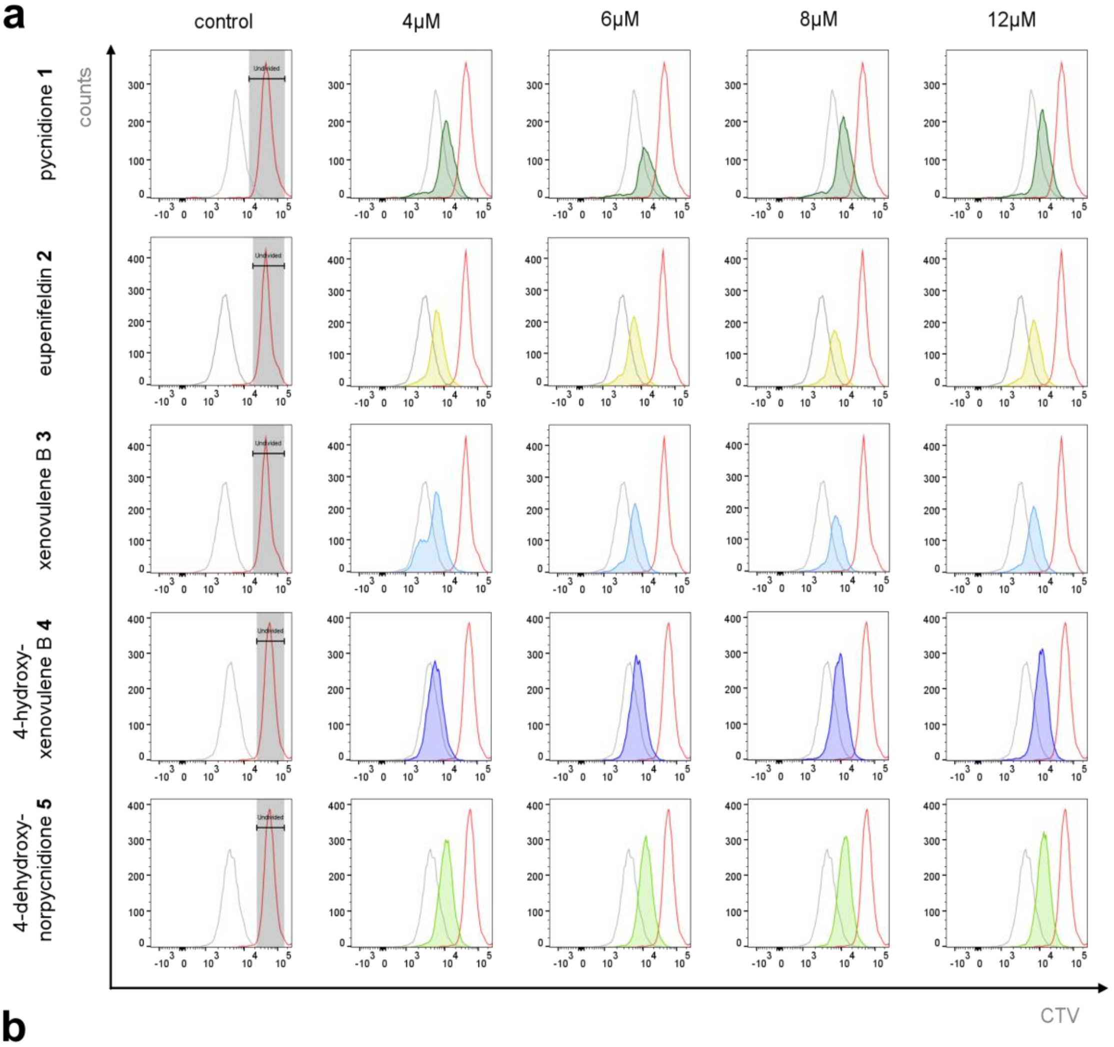

b

	MFI (a.u.)					fold change			
	0µM	*4µM*	*6µM*	*8µM*	*12µM*	*4µM*	*6µM*	*8µM*	*12µM*
pycnidione **1**	7124	12485	13732	12962	12985	1.75	1.93	1.82	1.82
eupenifeldin **2**	2946	6390	6689	6573	6747	2.17	2.27	2.23	2.29
xenovulene B **3**	3340	6937	6689	7281	7397	2.08	2.00	2.18	2.21
4-hydroxyxenovulene B **4**	4108	5664	6792	8736	10747	1.38	1.65	2.13	2.62
4-dehydroxy norpycnidione **5**	4091	10530	11534	12018	12496	2.57	2.82	2.94	3.05

Figure S 4. (a) Proliferation decrease of FAIK3-5 cells treated with 4 µM, 6 µM, 8 µM and 12 µM of tropolone-sesquiterpenoids (TS) (pycnidione **1**, eupenifeldin **2**, xenovulene B **3**, 4-hydroxyxenovulene B **4** and 4-dehydroxy norpycnidione **5**) for 24 h. Cell trace violet (CTV) stained cells were measured in a flow cytometer directly after staining to evaluate mean fluorescence intensities (MFI) of the undivided population (red) and 24 h after TS-compound treatment. MFI plots indicate a decrease of proliferation in samples treated with 4 µM – 12 µM of pycnidione **1** (dark green), eupenifeldin **2** (yellow), xenovulene B **3** (light blue) and 4-dehydroxy norpycnidione **5** (light green) assessed by consistently or increasingly higher MFI values compared to untreated controls (grey). In 4-hydroxyxenovulene B **4** (dark

blue) treated samples an enhanced proliferation inhibition with increasing compound concentrations was observed, with the highest fluorescence intensities at a concentration of 12 µM **(b)** Mean fluorescence (MFI) values and fold changes of FAIK3-5 cells treated with tropolone sesquiterpenoids (TS) (pycnidione **1**, eupenifeldin **2**, xenovulene B **3**, 4-hydroxyxenovulene B **4** and 4-dehydroxy norpycnidione **5**) in a concentration rage of 0 µM, 4 µM, 6 µM, 8 µM and 12 µM. MFI values of cell trace violet stained samples indicate proliferative behaviour of cell samples, whereby higher intensities imply a downshift of cell divisions. Measurements were conducted 24 h after compound treatment and fold changes were calculated with respect to untreated control samples (0 µM).

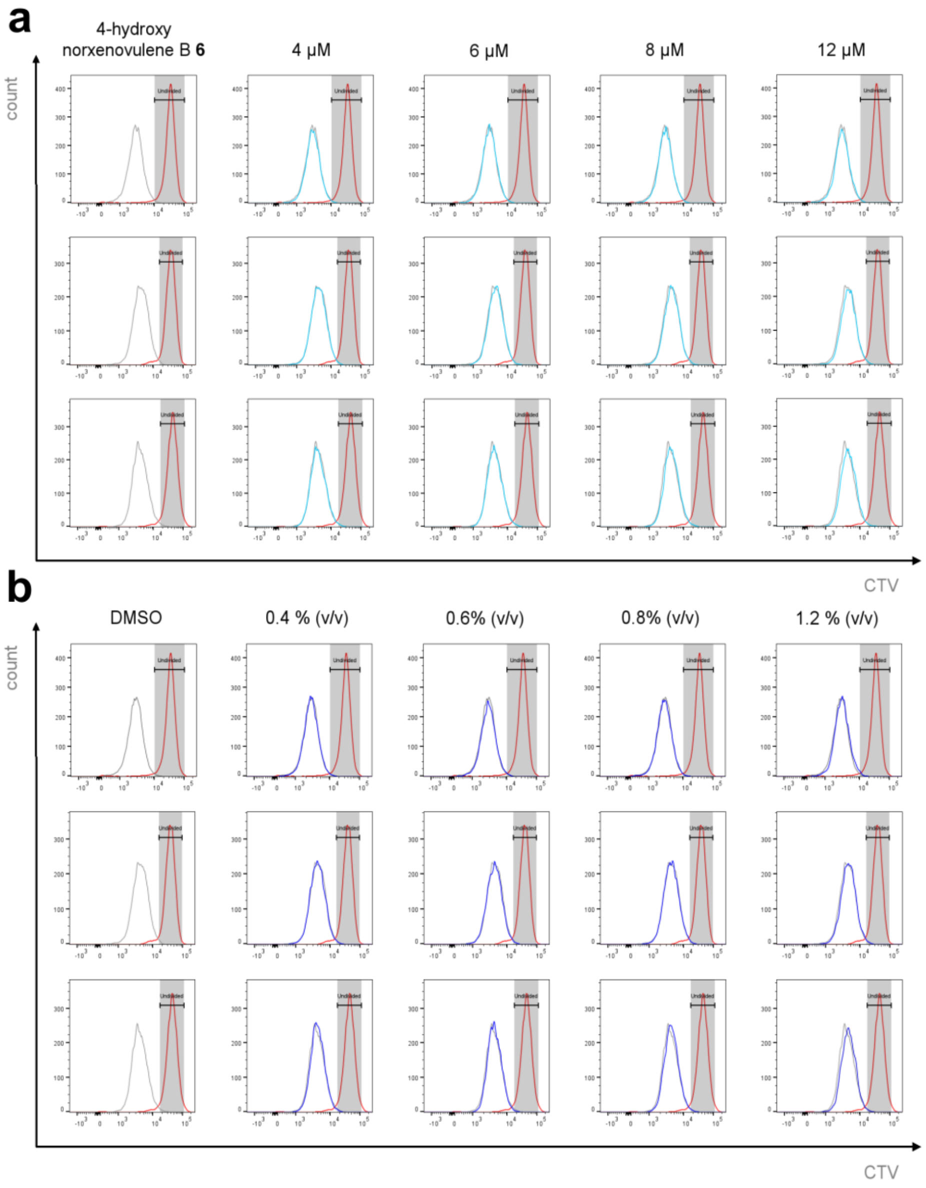

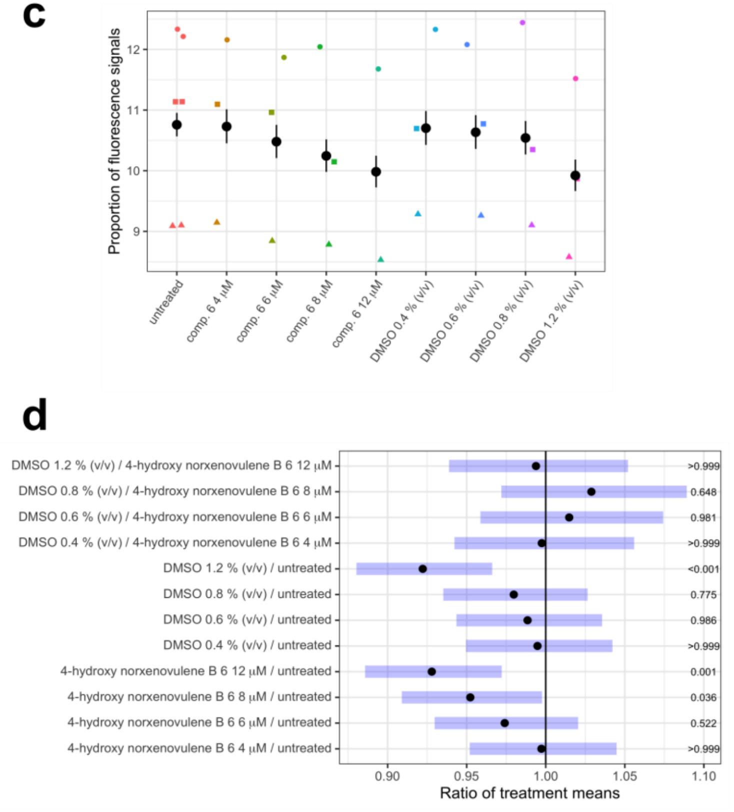

e

	MFI (a.u.)					fold change			
	0µM	*4µM*	*6µM*	*8µM*	*12µM*	*4µM*	*6µM*	*8µM*	*12µM*
4-hydroxy norxenovulene B **6** (1)	2723	2739	2804	2761	2849	1.01	1.03	1.01	1.05
4-hydroxy norxenovulene B **6** (2)	4066	4041	4185	4211	4334	0.99	1.03	1.04	1.07
4-hydroxy norxenovulene B **6** (3)	3808	3823	3870	4180	4254	1.00	1.02	1.10	1.12
	0% (v/v)	*0.4% (v/v)*	*0.6% (v/v)*	*0.8% (v/v)*	*1.2% (v/v)*	*0.4% (v/v)*	*0.6% (v/v)*	*0.8% (v/v)*	*1.2% (v/v)*
DMSO (1)	2697	2697	2755	2676	2888	1.00	1.02	0.99	1.07
DMSO (2)	4066	3983	3999	4066	4308	0.98	0.98	1.00	1.06
DMSO (3)	3808	3967	3942	4099	4299	1.04	1.04	1.08	1.13

Figure S 5 (Legend next page)

Figure S 5. Proliferation decrease of FAIK3-5 cells treated with the tropolone-lacking compound 4-hydroxy norxenovulene B **6** and respective concentrations of the vehicle control DMSO for 24 h. **(a, b)** Cell trace violet (CTV) stained cells were measured in a flow cytometer directly after staining to evaluate mean fluorescence intensities (MFI) of the undivided population (red) and 24 h after incubation with 4 µM, 6 µM, 8 µM and 12 µM of 4-hydroxy norxenovulene B **6** (light blue), 0.4% (v/v), 0.6% (v/v), 0.8% (v/v) and 1.2% (v/v) of the DMSO vehicle control (dark blue) and untreated controls (grey). **(c)** For statistical analysis obtained MFI values were normalized by calculating the proportions of measured fluorescence signals on d1 and d3. The graphic shows the observed proportions of fluorescence signals (y^1/y^3) for each treatment and their corresponding least square means (black dots) together with 95% confidence intervals. **(d)** Pairwise mean comparisons between 4-hydroxy norxenovulene B **6**, DMSO and the untreated control. The black line indicates the null hypothesis that the ratio between the average fluorescence signals (black dots) is one. The blue horizontal bars indicate simultaneous 95 % confidence intervals for the mean ratios. If a confidence interval encompasses the one, the ratio does not significantly differ from one. The numbers on the right side of the graphic are the p-values of the corresponding test (see statistical supplementary statS6). **(e)** Mean fluorescence (MFI) values and fold changes of FAIK3-5 cells treated with tropolone moiety lacking compound 4-hydroxy norxenovulene B **6** (N=3) and DMSO vehicle controls (N=3) in a concentration rage of 0 µM, 4 µM, 6 µM, 8 µM and 12 µM or respective volume percentages (0 % (v/v), 0.4 % (v/V), 0.6 % (v/v), 0.8 % (v/v) and 1.2 % (v/v). MFI values of cell trace violet stained samples indicate proliferative behavior of cell samples, whereby higher intensities imply a downshift of cell divisions. Measurements were conducted 24 h after compound treatment and fold changes were calculated with respect to untreated control samples (0 µM and 0 % (v/v)).

a

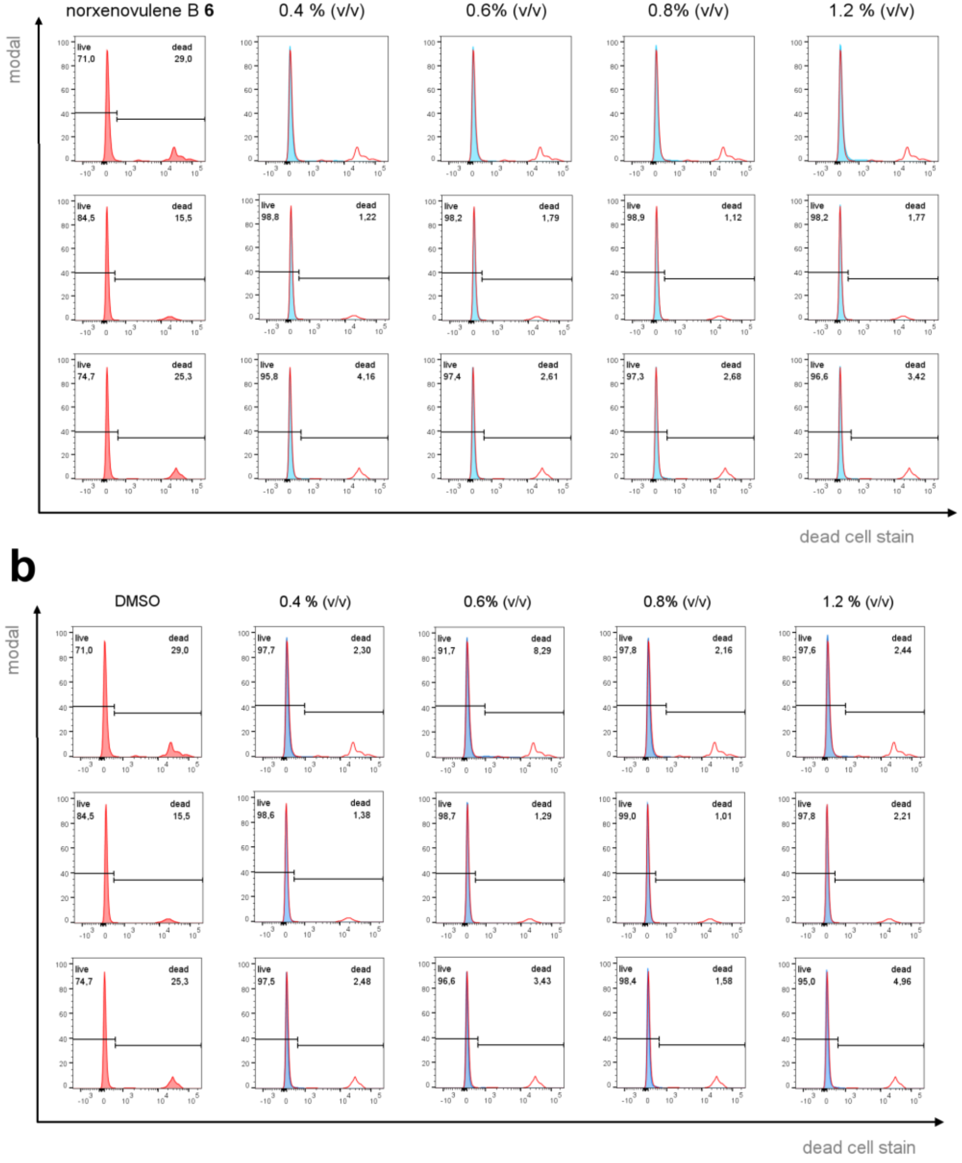

c

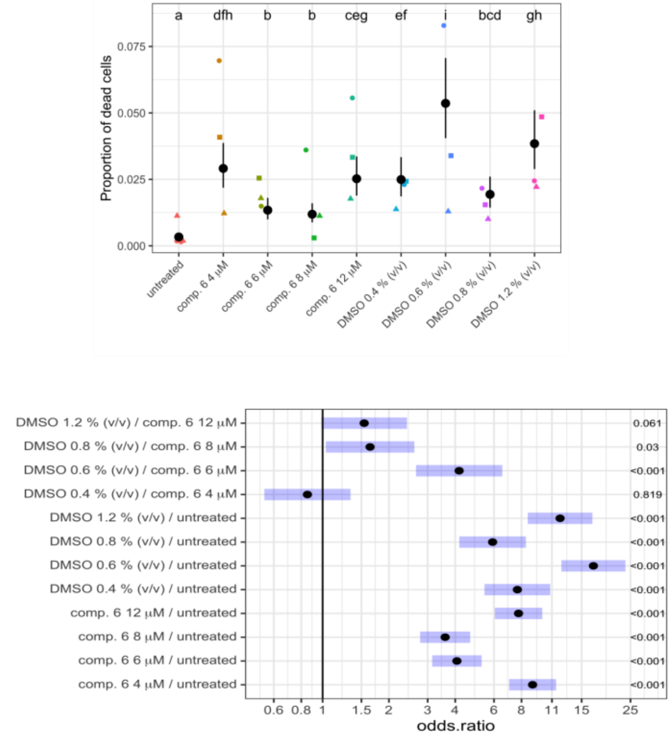

e

	cell viability [%]				
	0µM	*4µM*	*6µM*	*8µM*	*12µM*
pycnidione **1**	99.3	68.1	72.2	77.9	76.5
eupenifeldin **2**	99.8	72.1	69.4	55.8	66.8
xenovulene B **3**	99.9	92.9	92.9	89.8	89.8
4-hydroxyxenovulene B **4**	99.9	96.3	96.1	95.6	94.3
4-dehydroxy norpycnidione **5**	99.9	94.7	92.7	94.1	94.7
4-hydroxy norxenovulene B **6** (1)	99.8	93.0	98.5	96.4	94.4
4-hydroxy norxenovulene B **6** (2)	98.9	98.8	98.2	98.9	98.2
4-hydroxy norxenovulene B **6** (3)	99.7	95.8	97.4	97.3	96.6
	0% (v/v)	*0.4% (v/v)*	*0.6% (v/v)*	*0.8% (v/v)*	*1.2% (v/v)*
DMSO (1)	99.8	97.7	91.7	97.8	97.6
DMSO (2)	99.8	98.6	98.7	99.0	97.8
DMSO (3)	99.7	97.5	96.6	98.4	95.0

Figure S 6. (Legend next page)

Figure S 6. Assessment of cell viability of FAIK3-5 cells treated with **(a)** the tropolone moiety lacking compound 4-hydroxy norxenovulene B **6** and **(b)** DMSO vehicle control in a concentration rage of 0 µM, 4 µM, 6 µM, 8 µM and 12 µM or respective volume percentages (0 % (v/v), 0.4 % (v/v), 0.6 % (v/v), 0.8 % (v/v) and 1.2 % (v/v). Percentages of dead cells were obtained via SYTOX™ AADvanced™ dead cell staining and flow cytometric analysis of FAIK3-5 cells 24h after treatment with the compound and solvent controls. To set the threshold for dead cells' fluorescence intensities a living-dead control comprising a population of heat-killed FAIK3-5 cells was utilized and all samples were visualized in relation to the control (red). **(c)** Graphical overview of the data distribution of the proportion of dead cells in different experimental replicates (N=3) for each condition and the untreated control show model based means (black dots) and their 95% confidence intervals. Treatments marked with the same letter do not differ statistically significantly from each other ($\alpha = 0.05$). **(d)** Multiple comparison analysis was conducted based on odds-ratios of treatment means (black dots) that indicate the average chance to obtain dead cells in treatment conditions and the untreated control. Blue bars indicate simultaneous 95% confidence intervals of calculated ratios and p-values for each analysis are displayed on the ride side (see statistical supplementary statS8). (e) Cell viability of FAIK3-5 cells treated with tropolone sesquiterpenoids (TS) (pycnidione 1, eupenifeldin 2, xenovulene B 3, 4-hydroxyxenovulene B 4 and 4-dehydroxy norpycnidione 5), tropolone moiety lacking compound 4-hydroxy norxenovulene B 6 (N=3) and DMSO vehicle controls (N=3) in a concentration rage of 0 µM, 4 µM, 6 µM, 8 µM and 12 µM or respective volume percentages (0 % (v/v), 0.4 % (v/V), 0.6 % (v/v), 0.8 % (v/v) and 1.2 % (v/v). Percentages of cell viability were obtained via SYTOX™ AADvanced™ staining and subsequent flow cytometric analysis.

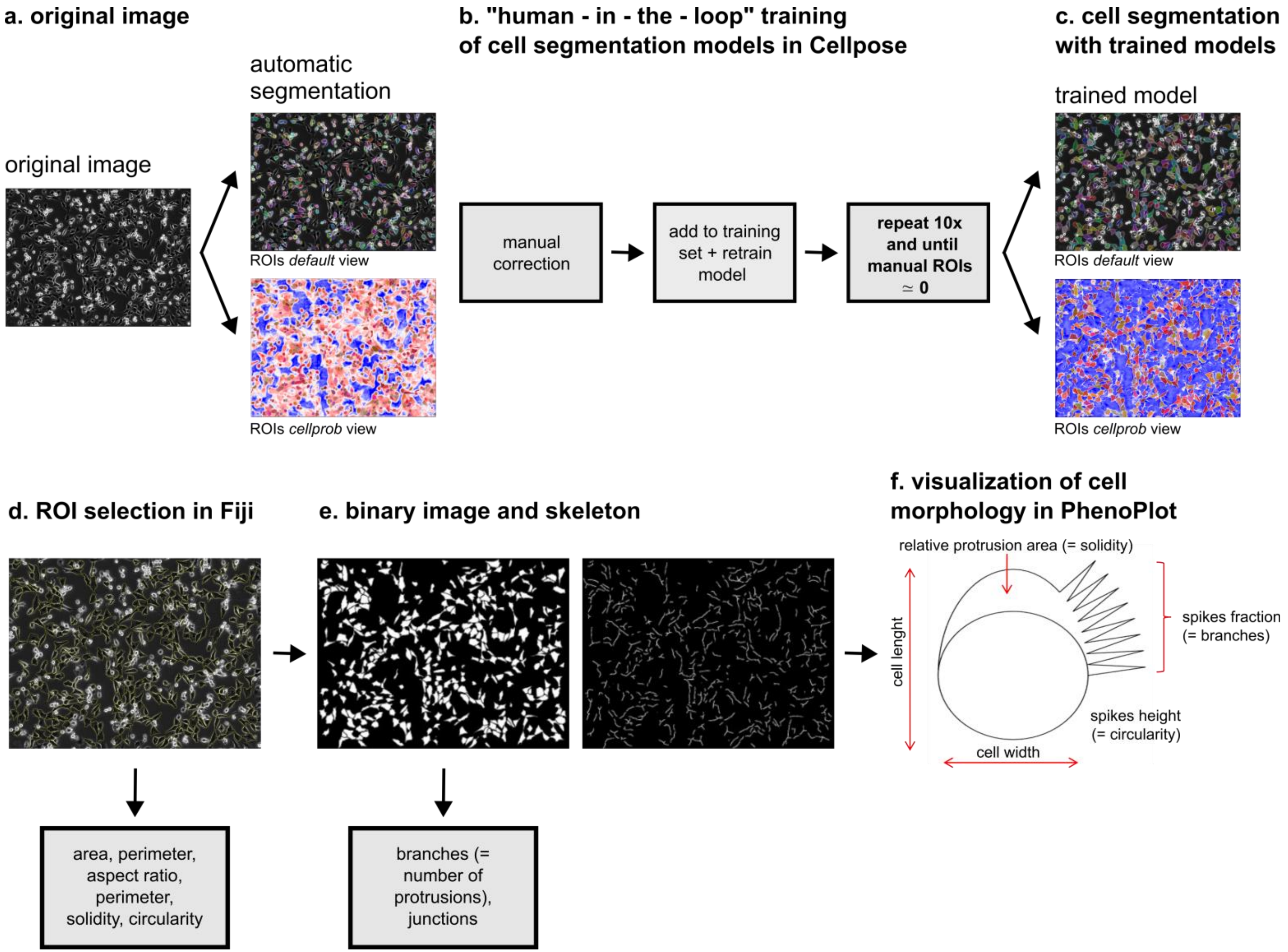

Figure S 7. (Legend next page)

Figure S 7. Workflow for analysis of morphometric parameters and ramification patterns. **(a)** For cell segmentation brightfield images were analyzed using the generalist deep-learning based algorithm *Cellpose* [1]. **(b)** Two cell segmentation models were trained to capture specific features of cell morphology accurately due to the high morphological variance between cells treated with bistropolones (**1** and **2**) (model 1) and those treated with monotropolons (**3**, **4** and **5**), the tropolone lacking compound **6** and untreated controls (model 2). This variance is caused by the increased occurrence of the telocyte-like subtype with nanotubes connecting the cells in the bistropolone samples. To operate *Cellpose* an Anaconda distribution (*Anaconda Software Distribution*. Vers. 2-2.4.0.) of Python (Version 3.8) [2] was used and a new Python environment for *Cellpose* was created in PowerShell Prompt (Version 0.0.1). (Further information for the installation of *Cellpose* can be found on https://github.com/MouseLand/cellpose). For the manual "human-in-the-loop" training of each model, we followed the same operational sequence on a minimum of 10 images. We continued until no further manual correction of the detected cells, referred to here as regions of interest (ROIs), was required. Firstly, we calibrated the cell diameter and then ran the default algorithm "*cyto2*" over the first training image. We used a flow threshold of 0.4, a cellprob threshold of 0.0, a stitch threshold of 0.0, and auto-adjusted the image saturation. ROIs were adjusted manually by removing incorrectly drawn ROIs and adding undetected ROIs to the image. To verify differences in cell morphology based on the cell's adjustments to TS compounds and controls, only living, adherent cells were included in the analysis and the models were trained to exclude oversaturated, floating cells. **(c)** For the final analysis the trained models were run on images of FAIK3-5 cells treated with the investigated compounds (**1**–**6**) and the untreated control of three independent experiments (N=3), without further manual correction of the detected ROIs. To ensure accurate measurements, we only included complete cells in our analysis. Therefore, we excluded ROIs that detected cells cut off by the image edges or overlapping regions. **(d)** Detected ROIs were transferred to Fiji (ImageJ Version 1.53c bundled with Java 1.8.0_172) [3], an overlay with the original image was created to check correct ROI selection and measurement of cell area, shape descriptors (aspect ratio, circularity, roundness and solidity), perimeter and was conducted. **(f)** For the analysis of ramification patterns, selected ROIs of separated cellular shapes were used to create a binary image, on which the "Skeletonize 2D/3D" and "Analyze Skeleton (2D/3D)" [4] plugins were run for visualization and calculation of e.g. branch numbers (via connection of end-points, end-

points and junctions, or junctions and junctions) and number of branch junctions. The obtained values for geometrical features, shape descriptors and branch numbers were normalized to the highest obtained mean value of each analysed parameter for subsequent visualization in PhenoPlot [5].

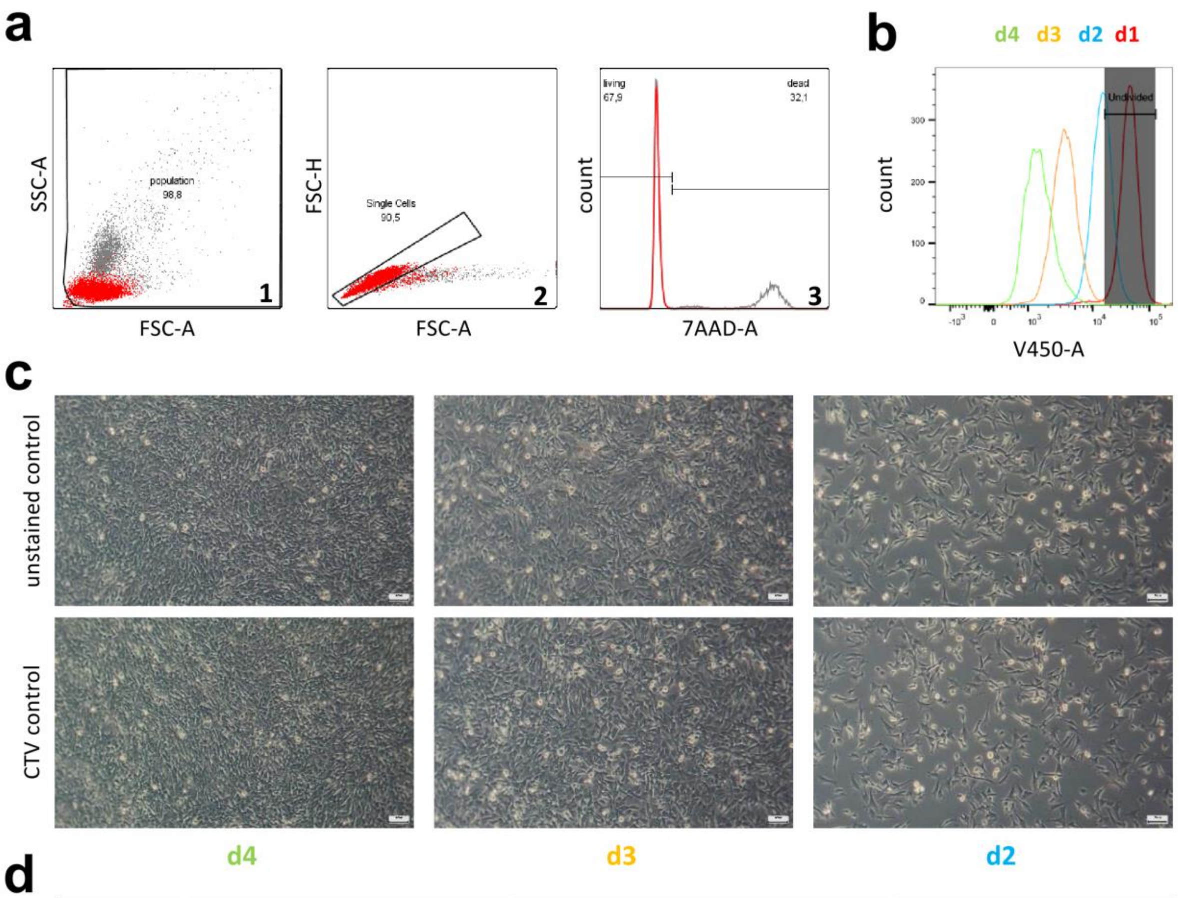

d

	mean fluorescence intensity	counted living cells	viability [%]
**d1**	46147	8393	91.7
**d2**	15444	9535	97.0
**d3**	3936	9432	97.4
**d4**	1693	9482	98.3

Figure S 8. Gating strategy for cytotoxicity assessment and proliferation analysis and preliminary tests on FAIK3-5 cells to assess optimal experimental conditions for subsequent proliferation analysis. **(a)** Backgating plots of the living cell population show the strategy for living/dead discrimination in a living/dead control sample comprising 50 % of heat-killed FAIK3-5 cells. After out-gating of (1) cell debris and (2) doublets, the (3) living and dead population is visualized in a histogram showing fluorescence intensities of Sytox AADvanced stained cells (7AAD-A channel). Thereafter, the threshold for dead cells' fluorescence intensities was set in the living/dead control and assigned to the remaining samples. **(b)** For

proliferation analysis the mean fluorescence intensity (MFI) of cell trace violet (CTV, measured in V450-A channel) of samples on d1 was set as undivided population in the living cell population of the analysed samples. To assess the optimal staining period for proliferation tests, CTV intensities and cell viability of stained FAIK3-5 cells were analysed on three subsequent days. **(c, d)** Proliferative behavior of FAIK3-5 cells was evaluated by normalizing the measured fluorescence intensities at day 2 - 4 to their respective MFIs of the undivided population at d1 of the experiment. As the cells continuously proliferate (**c**), the intensity of CTV decreases with each cell division, while cell viability remains over 90 % (d). For CTV staining and living/dead analysis 10 000 events per sample were counted.

References

1. Stringer, C., Wang, T., Michaelos, M., and Pachitariu, M. (2021). Cellpose: a generalist algorithm for cellular segmentation. Nature methods *18,* 100–106. 10.1038/s41592-020-01018-x.
2. Python Programming Language (2007).
3. Schindelin, J., Arganda-Carreras, I., Frise, E., Kaynig, V., Longair, M., Pietzsch, T., Preibisch, S., Rueden, C., Saalfeld, S., and Schmid, B., et al. (2012). Fiji: an open-source platform for biological-image analysis. Nature methods *9,* 676–682. 10.1038/nmeth.2019.
4. Arganda-Carreras, I., Fernández-González, R., Muñoz-Barrutia, A., and Ortiz-De-Solorzano, C. (2010). 3D reconstruction of histological sections: Application to mammary gland tissue. Microscopy research and technique *73,* 1019–1029. 10.1002/jemt.20829.
5. Sailem, H.Z., Sero, J.E., and Bakal, C. (2015). Visualizing cellular imaging data using PhenoPlot. Nat Commun *6,* 5825. 10.1038/ncomms6825.

Statistical Supplementary Information

Contents

Statistical supplementary statS1: Estimations of IC50

Data 22

Hierarchical non linear modelling 22

FAIK3-5 22

Jurkat 24

MDA-MD 231 25

PC-3 26

Statistical supplementary statS2: Proteins

Overview about the data 28

Statistical model 29

Estimated variance components 29

Statistical supplementary statS3: Proteins after 5-Azacytidine treatment

Overview about the data 31

Statistical model 31

Estimated variance components 32

Model based least square means and their 95 % confidence intervals 32

Mean comparisons 32

Statistical supplementary statS4: Cell morphology

Packages used for evaluation 34

Overview about the data 34

Modeling 35

Multiple comparisons 36

Graphical overview about the multiple comparisons 38

Statistical supplementary statS5: Branches

Packages used for evaluation 39

Overview about the data 39

Modeling 40

Multiple comparisons 40

Graphical overview about the multiple comparisons 42

Statistical supplementary statS6: Proliferation for 4-hydroxy norxenovulene B 6 and DMSO
Data set 43
Statistical model 44
Model based means and their pointwise 95% confidence intervals 44
Mean comparisons (contrast tests) 44
Statistical supplementary statS7: Proliferation
Data set 47
Linear model 47
Analysis of variance 48
Model based least square means and their pointwise 95% confidence intervals 48
Mean comparisons (contrast tests) 48
Statistical supplementary statS8: Toxicity for 4-hydroxy norxenovulene B 6 and DMSO
Data set 51
Statistical model 52
Model based means and their pointwise 95% confidence intervals 52
Multiple comparisons 52
Statistical supplementary statS9: Toxicity
Data set 55
Statistical model 55
Deviance analysis 56
Estimated average proportions and their comparisons 56
Estimated mean proportions of dead cells 56
Mean comparisons (contrast tests) 57

Statistical supplementary statS1: Estimations of IC50

Bergmann et al. 2024

Data

```
# First rows of the data
head(dat)
```

```
##      line     compound repetition  conc  mean_int
## 1 FAIK3_5 pycnidione_1          1 0e+00 17218.000
## 2 FAIK3_5 pycnidione_1          1 1e-03 19811.667
## 3 FAIK3_5 pycnidione_1          1 1e-02 20228.667
## 4 FAIK3_5 pycnidione_1          1 1e-01  8383.333
## 5 FAIK3_5 pycnidione_1          1 1e+00  7260.333
## 6 FAIK3_5 pycnidione_1          1 1e+01  4559.333
```

- line: Indecator for the cell lines
- compound: Indecator for the six compounds and DMSO without a compound as control
- repetition: Indecator wich experiment belongs to the specific repetition (three repetitions in all)
- mean_int: Mean fluorescence intensity, average over 12 observations (four per each of three wells)
- conc: Concentration

Hierarchical non linear modelling

For each cell line, the IC50s of the compounds were estimated based on hierarchical nonlinear regression models. All fitted models were based on the four-parameter log-logistic function with parameters

- b: Steepness of the curve at IC50
- c: Lower asymptote
- e: IC50
- d: Upper asymptote

In each of the fitted models (one for each cell line), the lower asymptote was estimated from all observations, regardless to which compound they belong. For MDA-MD 231 cells, also the steepness parameter was held fixed for all compounds (due to convergance problems), but e, d were allowed to differ between the compounds. For the other three cell lines, the parameters b, e, d were allowed to differ between the compounds. For all four models, the parameters d and e were modelled as random effects.

IC50s were derived from the fitted models and for each cell line, the estimated IC50s for the compounds were tested against the IC50 of DMSO in a Dunnett type test procedure (p-value adjustment following Hothoern et al. 2008).

FAIK3-5

```
# Subset for FAIK3-5 cells
dat_faik <- filter(dat, line=="FAIK3_5", compound != "DMSO_without_cells")
%>%
```

```
        mutate(comrep=interaction(compound, repetition)) %>%
        select(mean_int, conc, comrep, compound) %>%
        droplevels() %>%
        data.frame()

# Hierarchical nonlinear model
fit_faik <- medrm(mean_int~conc,
                  data=dat_faik,
                  fct=LL.4(),
                  curveid=b + e + d ~ compound,
                  random= d + e ~ 1|comrep)

# Estimated IC50s, their standard errors and 95% confidence intervals (lowe
r, upper)
as.data.frame(ED(fit_faik, 50, interval="delta", display = FALSE))

##                                    Estimate Std. Error       Lower
Upper
## e:4_dehydroxy_norpycnidione_5:50  1.4648800 0.44754053  0.58174587  2.34
80142
## e:4_hydrox_xenovulene_B_4:50      6.4576130 1.62448228  3.25201326  9.66
32127
## e:4_hydroxy_norxenovulene_B_6:50 22.7257179 2.62369080 17.54837461 27.90
30612
## e:DMSO_with_cells:50             22.9778857 2.48126862 18.08158488 27.87
41866
## e:eupenifeldin_2:50               0.1278044 0.05808026  0.01319430  0.24
24145
## e:pycnidione_1:50                 0.1973568 0.09219915  0.01541975  0.37
92939
## e:xenovulene_B_3:50               2.8011319 0.84234664  1.13892467  4.46
33391

# Dunnett test of the IC50s

ED_multcomp <- ED(fit_faik,
                  50,
                  interval="delta",
                  multcomp = TRUE,
                  display=FALSE)

nvec <- 1:7
names(nvec) <- names(ED_multcomp$EDmultcomp$coef)

summary(glht(ED_multcomp[["EDmultcomp"]],
                               linfct=contrMat(nvec,
                                               "Dunnett",
                                               base=4)))

##    lhs1 rhs     estimate        lwr        upr
## 1   5-7   0 -21.5130057 -27.389563 -15.636449
## 2   4-7   0 -16.5202728 -23.428357  -9.612189
## 3   6-7   0  -0.2521678  -8.532694   8.028358
## 4   2-7   0 -22.8500813 -28.675879 -17.024283
## 5   1-7   0 -22.7805289 -28.605027 -16.956030
## 6   3-7   0 -20.1767539 -26.245270 -14.108238
```

Jurkat

```
# Subset for Jurkat cells
dat_jurkat <- filter(dat, line=="Jurkat", compound != "DMSO_without_cells")
 %>%
        mutate(comrep=interaction(compound, repetition)) %>%
        select(mean_int, conc, comrep, compound) %>%
        droplevels() %>%
        data.frame()

# Hierarchical nonlinear model
fit_jurkat <- medrm(mean_int~conc,
                    data=dat_jurkat,
                    fct=LL.4(),
                    curveid=b + e + d ~ compound,
                    random= d + e ~ 1|comrep)

# Estimated IC50s, their standard errors and 95% confidence intervals (lowe
r, upper)
as.data.frame(ED(fit_jurkat, 50, interval="delta", display = FALSE))

##                                      Estimate Std. Error       Lower
Upper
## e:4_dehydroxy_norpycnidione_5:50  3.2130030 0.62143604  1.98671986  4.43
92861
## e:4_hydrox_xenovulene_B_4:50     10.5252475 0.87274227  8.80306043 12.24
74346
## e:4_hydroxy_norxenovulene_B_6:50 21.5883406 1.45271227 18.72169554 24.45
49856
## e:DMSO_with_cells:50             17.1239168 1.67252971 13.82350480 20.42
43287
## e:eupenifeldin_2:50               0.0689933 0.03927945 -0.00851705  0.14
65037
## e:pycnidione_1:50                 0.1957781 0.06811034  0.06137559  0.33
01806
## e:xenovulene_B_3:50               2.6283387 0.63275187  1.37972599  3.87
69514

# Dunnett test of the IC50s

ED_multcomp <- ED(fit_jurkat,
                  50,
                  interval="delta",
                  multcomp = TRUE,
                  display=FALSE)

nvec <- 1:7
names(nvec) <- names(ED_multcomp$EDmultcomp$coef)

summary(glht(ED_multcomp[["EDmultcomp"]],
                                linfct=contrMat(nvec,
                                                "Dunnett",
                                                base=4)))

##   lhs1 rhs   estimate         lwr        upr
## 1  5-7   0 -13.910914 -18.0571301  -9.764697
```

```
## 2  4-7   0  -6.598669 -10.9864917  -2.210847
## 3  6-7   0   4.464424  -0.6890397   9.617887
## 4  2-7   0 -17.054923 -20.9775078 -13.132339
## 5  1-7   0 -16.928139 -20.8502719 -13.006005
## 6  3-7   0 -14.495578 -18.6441720 -10.346984
```

MDA-MD 231

```
# Subset for MDA-MD 231 cells
dat_mda_md <- filter(dat, line=="MDA_MD_231", compound != "DMSO_without_cel
ls") %>%
        mutate(comrep=interaction(compound, repetition)) %>%
        select(mean_int, conc, comrep, compound) %>%
        droplevels() %>%
        data.frame()

# Hierarchical nonlinear model
fit_mda_md <- medrm(mean_int~conc,
                    data=dat_mda_md,
                    fct=LL.4(),
                    curveid= e + d ~ compound,
                    random= d + e ~ 1|comrep)

## Warning in nlme.formula(mean_int ~ meLL.4(conc, b, c, d, e), fixed = lis
t(b ~ :
## Iteration 1, LME step: nlminb() did not converge (code = 1). Do increase
## 'msMaxIter'!

# Estimated IC50s, their standard errors and 95% confidence intervals (lowe
r, upper)
as.data.frame(ED(fit_mda_md, 50, interval="delta", display = FALSE))

##                                    Estimate Std. Error    Lower    Upper
## e:4_dehydroxy_norpycnidione_5:50 16.28259   2.804129 10.74918 21.81599
## e:4_hydrox_xenovulene_B_4:50     18.41708   3.328798 11.84835 24.98582
## e:4_hydroxy_norxenovulene_B_6:50 17.78042   2.965391 11.92879 23.63204
## e:DMSO_with_cells:50             20.29586   3.337690 13.70958 26.88214
## e:eupenifeldin_2:50              16.44847   3.144468 10.24348 22.65347
## e:pycnidione_1:50                16.64705   3.143708 10.44355 22.85055
## e:xenovulene_B_3:50              18.58067   3.309810 12.04940 25.11194

# Dunnett test of the IC50s

ED_multcomp <- ED(fit_mda_md,
                  50,
                  interval="delta",
                  multcomp = TRUE,
                  display=FALSE)

nvec <- 1:7
names(nvec) <- names(ED_multcomp$EDmultcomp$coef)

summary(glht(ED_multcomp[["EDmultcomp"]],
                                linfct=contrMat(nvec,
                                                "Dunnett",
                                                base=4)))
```

```
##   lhs1 rhs  estimate       lwr      upr
## 1  5-7   0 -4.013272 -14.37244 6.345894
## 2  4-7   0 -1.878778 -13.00179 9.244231
## 3  6-7   0 -2.515441 -13.11113 8.080248
## 4  2-7   0 -3.847386 -14.67960 6.984828
## 5  1-7   0 -3.648808 -14.48749 7.189874
## 6  3-7   0 -1.715189 -12.81746 9.387079
```

PC-3

```
# Subset for Jurkat cells
dat_pc3 <- filter(dat, line=="PC_3", compound != "DMSO_without_cells") %>%
        mutate(comrep=interaction(compound, repetition)) %>%
        select(mean_int, conc, comrep, compound) %>%
        droplevels() %>%
        data.frame()

# Hierarchical nonlinear model
fit_pc3 <- medrm(mean_int~conc,
                   data=dat_pc3,
                   fct=LL.4(),
                   curveid=b + e + d ~ compound,
                   random= d + e ~ 1|comrep)

# Estimated IC50s, their standard errors and 95% confidence intervals (lowe
r, upper)
as.data.frame(ED(fit_pc3, 50, interval="delta", display = FALSE))

##                                    Estimate Std. Error     Lower     Uppe
r
## e:4_dehydroxy_norpycnidione_5:50  5.041838   1.231958  2.610808  7.47286
8
## e:4_hydrox_xenovulene_B_4:50     14.387172   2.112586 10.218394 18.55595
0
## e:4_hydroxy_norxenovulene_B_6:50 15.182357   1.716479 11.795220 18.56949
4
## e:DMSO_with_cells:50             14.722614   1.792643 11.185182 18.26004
6
## e:eupenifeldin_2:50               7.605650   1.662656  4.324722 10.88657
8
## e:pycnidione_1:50                 6.257120   1.469021  3.358292  9.15594
8
## e:xenovulene_B_3:50               8.610989   1.717159  5.222510 11.99946
7

# Dunnett test of the IC50s

ED_multcomp <- ED(fit_pc3,
                  50,
                  interval="delta",
                  multcomp = TRUE,
                  display=FALSE)

nvec <- 1:7
names(nvec) <- names(ED_multcomp$EDmultcomp$coef)
```

```
summary(glht(ED_multcomp[["EDmultcomp"]],
                                 linfct=contrMat(nvec,
                                                 "Dunnett",
                                                 base=4)))
```

```
##   lhs1 rhs   estimate        lwr         upr
## 1  5-7   0 -9.6807758 -15.075650 -4.2859020
## 2  4-7   0 -0.3354424  -7.199931  6.5290457
## 3  6-7   0  0.4597432  -5.661179  6.5806654
## 4  2-7   0 -7.1169640 -13.164776 -1.0691518
## 5  1-7   0 -8.4654941 -14.198617 -2.7323707
## 6  3-7   0 -6.1116252 -12.265527  0.0422762
```

Statistical supplementary statS2: Proteins

Bergmann et al. 2024

```
# Load the packages used for evaluation
library(tidyverse)
library(lme4)
library(lmerTest)
library(tidyverse)
library(emmeans)
```

Overview about the data

Description of the variables

- Rep: Replication
- Plate: Plate in each replication
- Well: Well in each plate in each replication
- Compund: Chemical compounds (treatment variable)
- Protein: Protein content in pg/ml

```
# First six rows of the data set
head(prote)

##   Rep Plate Well       Compound  Protein
## 1   1     1    1   pycnidione 1 5.819760
## 2   1     1    1   pycnidione 1 4.158405
## 3   1     1    1   pycnidione 1 4.302978
## 4   1     1    2 xenovulene B 3 7.161895
## 5   1     1    2 xenovulene B 3 6.590697
## 6   1     1    2 xenovulene B 3 7.172868

# 69 observations at all (rows)
str(prote)

## 'data.frame':    69 obs. of  5 variables:
##  $ Rep     : Factor w/ 3 levels "1","2","3": 1 1 1 1 1 1 1 1 1 1 ...
##  $ Plate   : Factor w/ 2 levels "1","2": 1 1 1 1 1 1 1 1 1 1 ...
##  $ Well    : Factor w/ 6 levels "1","2","3","4",..: 1 1 1 2 2 2 3 3 3 4
...
##  $ Compound: Factor w/ 8 levels "DMSO 0.6 % (v/v)",..: 3 3 3 5 5 5 4 4 4
 7 ...
##  $ Protein : num  5.82 4.16 4.3 7.16 6.59 ...
```

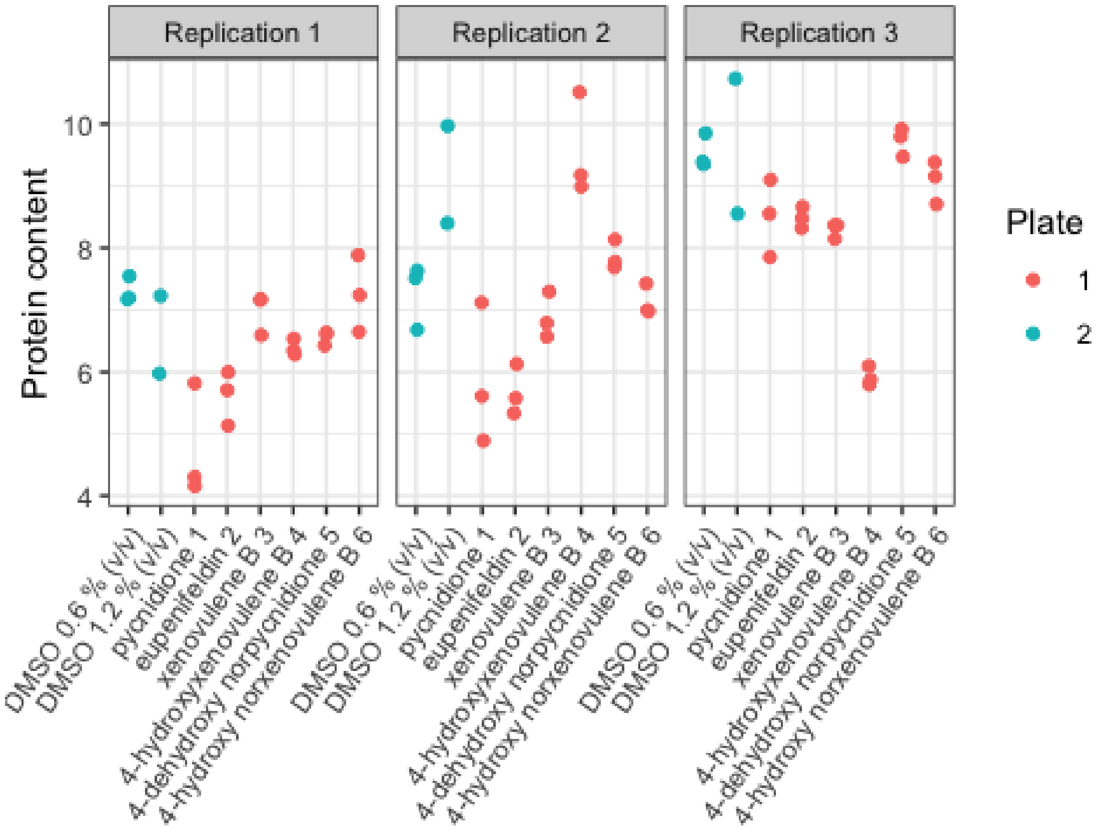

Statistical model

Hierarchical experimental design, since the plates are nested in the replications and the wells are nested in each plate.

- Linear mixed model
- Protein as response variable
- Compounds as fixed effects
- Rep, Plates and Wells as random effects

```
fit <- lmer(Protein ~ Compound + (1|Rep) + (1|Rep:Plate) + (1|Rep:Plate:Wel
l),
            data=prote)
```

Estimated variance components

```
print(VarCorr(fit),comp=c("Variance"),digits=2)

##  Groups         Name        Variance
##  Rep:Plate:Well (Intercept) 1.2e+00
##  Rep:Plate      (Intercept) 1.5e-09
##  Rep            (Intercept) 9.7e-01
##  Residual                   4.0e-01
```

- Most of the total variance can be explained by the wells.
- The plates do not explain any variance

```
anova(fit,
      ddf="Kenward-Roger")
```

```
## Type III Analysis of Variance Table with Kenward-Roger's method
##          Sum Sq Mean Sq NumDF  DenDF F value Pr(>F)
## Compound 3.3223 0.47462     7 10.514  1.1038 0.4261
```

- The p-value of 0.4261 is bigger than $\alpha = 0.05$
- No different mean protein contents between the compounds

Statistical supplementary statS3: Proteins after 5-Azacytidine treatment

Bergmann et al. 2024

```
# Load the packages used for evaluation
library(tidyverse)
library(lme4)
library(lmerTest)
library(tidyverse)
library(emmeans)
library(multcomp)
```

Overview about the data

Description of the variables

- Rep: Replication
- Plate: Plate in each replication
- Well: Well in each plate in each replication
- Compund: Chemical compounds (treatment variable)
- Protein: Protein content in pg/ml

```
# First six rows of the data set
head(aza)

##   Rep Plate Well       Compound  Protein
## 1   1     1    1   pycnidione 1 22.10247
## 2   1     1    1   pycnidione 1 19.06987
## 3   1     1    1   pycnidione 1 18.17886
## 4   1     1    2 xenovulene B 3 17.96557
## 5   1     1    2 xenovulene B 3 14.10570
## 6   1     1    2 xenovulene B 3 17.36827

# 72 observations at all (rows)
str(aza)

## 'data.frame':    72 obs. of  5 variables:
##  $ Rep     : Factor w/ 3 levels "1","2","3": 1 1 1 1 1 1 1 1 1 1 ...
##  $ Plate   : Factor w/ 2 levels "1","2": 1 1 1 1 1 1 1 1 1 1 ...
##  $ Well    : Factor w/ 6 levels "1","2","3","4",..: 1 1 1 2 2 2 3 3 3 4
...
##  $ Compound: Factor w/ 8 levels "DMSO 0.6 % (v/v)",..: 3 3 3 5 5 5 4 4 4
 7 ...
##  $ Protein : num  22.1 19.1 18.2 18 14.1 ...
```

Statistical model

Hierarchical experimental design, since the plates are nested in the replications and the wells are nested in each plate.

- Linear mixed model
- Protein as response variable
- Compounds as fixed effects
- Rep, Plates and Wells as random effects

```
fit <- lmer(Protein ~ Compound + (1|Rep) + (1|Rep:Plate) + (1|Rep:Plate:Wel
l),
            data=aza)
```

Estimated variance components

```
print(VarCorr(fit),comp=c("Variance"),digits=2)

##  Groups          Name        Variance
##  Rep:Plate:Well (Intercept) 0.26
##  Rep:Plate       (Intercept) 0.24
##  Rep             (Intercept) 0.13
##  Residual                    3.96
```

- The residual variance is relatively high, compared to the variance that can be explained due to the experimental design.

```
anova(fit,
      ddf="Kenward-Roger")

## Type III Analysis of Variance Table with Kenward-Roger's method
##          Sum Sq Mean Sq NumDF  DenDF F value   Pr(>F)
## Compound 160.94  22.991     7 10.845  5.3773 0.007245 **
## ---
## Signif. codes:  0 '***' 0.001 '**' 0.01 '*' 0.05 '.' 0.1 ' ' 1
```

- The p-value of 0.007245 is smaller than $\alpha = 0.05$

Model based least square means and their 95 % confidence intervals

```
means <- emmeans(fit,
                 specs="Compound",
                 adjust="mvt",
                 type="response")

means

##  Compound                        emmean    SE   df lower.CL upper.CL
##  DMSO 0.6 % (v/v)                  16.9 0.807 14.5     14.4     19.5
##  DMSO 1.2 % (v/v)                  18.9 0.807 14.5     16.3     21.4
##  pycnidione 1                      17.8 0.807 14.5     15.3     20.4
##  eupenifeldin 2                    13.4 0.807 14.5     10.9     15.9
##  xenovulene B 3                    15.1 0.807 14.5     12.5     17.6
##  4-hydroxyxenovulene B 4           14.0 0.807 14.5     11.4     16.5
##  4-dehydroxy norpycnidione 5       15.0 0.807 14.5     12.4     17.5
##  4-hydroxy norxenovulene B 6       15.5 0.807 14.5     13.0     18.1
##
## Degrees-of-freedom method: kenward-roger
## Confidence level used: 0.95
## Conf-level adjustment: mvt method for 8 estimates
```

A graphical overview about this table is given in fig. 2 c.

Mean comparisons

Only the mean comparisons defined in the list below were tested

```
cont_list
```

```
## $`DMSO 0.6 % (v/v) - pycnidione 1`
## [1]  1  0 -1  0  0  0  0  0
##
## $`DMSO 0.6 % (v/v) - eupenifeldin 2`
## [1]  1  0  0 -1  0  0  0  0
##
## $`DMSO 0.6 % (v/v) - xenovulene B 3`
## [1]  1  0  0  0 -1  0  0  0
##
## $`DMSO 0.6 % (v/v) - 4-hydroxy norxenovulene B 6`
## [1]  1  0  0  0  0  0  0 -1
##
## $`DMSO 1.2 % (v/v) - 4-hydroxyxenovulene B 4`
## [1]  0  1  0  0  0 -1  0  0
##
## $`DMSO 1.2 % (v/v) - 4-dehydroxy norpycnidione 5`
## [1]  0  1  0  0  0  0 -1  0

comp <- contrast(means,
                 method=cont_list,
                 adjust="mvt")
```

```
comp

##                                          contrast   estimate p.value   si
g
## 1 DMSO 0.6 % (v/v) - 4-hydroxy norxenovulene B 6  1.4027470   0.669 FALS
E
## 2                DMSO 0.6 % (v/v) - eupenifeldin 2  3.5509246   0.046  TRU
E
## 3                  DMSO 0.6 % (v/v) - pycnidione 1 -0.8899113   0.918 FALS
E
## 4                DMSO 0.6 % (v/v) - xenovulene B 3  1.8763319   0.418 FALS
E
## 5 DMSO 1.2 % (v/v) - 4-dehydroxy norpycnidione 5  3.9179278   0.028  TRU
E
## 6      DMSO 1.2 % (v/v) - 4-hydroxyxenovulene B 4  4.9004060   0.008  TRU
E
```

A graphical overview about this table is given in fig. 2 d.

Statistical supplementary statS4: Cell morphology

Bergmann et al. 2024

Packages used for evaluation

```
library(tidyverse)
library(emmeans)
library(lme4)
library(multcomp)
library(tukeytrend)
library(ggpubr)
```

Overview about the data

The complete experiment was run in three separate repetitions (Variable trial). For treatment (six compounds plus untreated control), cells were cultured on one well per treatment within each of the trials. For each well, several morphological characteristics were measured on several hundreds of cells (variable n) resulting in a total of 16632 analysed cells. Since several of the measurements resulted in highly right or left skewed observations that were impossible to center by transformation, modelling was done based on the average measurement per well.
This data sets (dat) is depicted below and contains the following variables:

- Comp_ID: ID for the compounds of interest (see below)
- Trial: Indicator variable for the three repetitions
- Well: Indicator variable for the wells in each repetition
- Area_m: Average cell area per well
- Perim_m: Average perimeter per well
- AR_m: Average aspect ratio per well
- Circ_m: Average circularity per well
- Solidity_m: Average solidity per well
- n: Number of cells per well

Compound IDs

- 1 = pycnidione
- 2 = eupenifeldin
- 3 = xenovulene B
- 4 = 4-hydroxyxenovulene B
- 5 = 4-dehydroxy norpycnidione
- 6 = 4-hydroxy norxenovulene B
- U = Untreated

```
dat

##    Comp_ID Trial Well    Area_m   Perim_m     AR_m    Circ_m Solidity_m
   n
## 1        1     1    1  670.6581 127.58322 2.092708 0.5236193  0.8185791
 373
## 2        2     1    2  791.9424 151.75865 1.955226 0.4436452  0.7430968
 310
## 3        3     1    3  975.8344 139.81239 1.913272 0.6078634  0.8770748
 615
```

```
## 4          4     1     4 1048.7376 151.33379 2.057143 0.5621454  0.8521684
 392
## 5          5     1     5  773.6091 130.99050 2.283691 0.5629228  0.8659365
 803
## 6          6     1     6  560.7641 103.78866 1.816892 0.6410123  0.8926472
1301
## 7          U     1     7  490.0072  97.16836 1.840930 0.6410694  0.8923834
1484
## 8          1     2     8  529.2838 117.34503 1.961732 0.4868156  0.7694134
 358
## 9          2     2     9  541.9511 115.49518 2.010395 0.5094737  0.7923684
 228
## 10         3     2    10  832.7332 130.05037 1.912398 0.6061806  0.8706806
 576
## 11         4     2    11 1046.1742 142.11862 1.718805 0.6384778  0.8894157
 676
## 12         5     2    12  700.1952 129.86816 2.498716 0.5276609  0.8444358
 693
## 13         6     2    13  481.7690  97.00257 1.886395 0.6352602  0.8901686
1518
## 14         U     2    14  396.9946  87.20479 1.816864 0.6458562  0.8904478
1773
## 15         1     3    15  489.1608 109.31691 2.004433 0.5105155  0.7915464
 194
## 16         2     3    16  529.8521 115.10853 2.021799 0.5102360  0.7929794
 339
## 17         3     3    17  838.6017 130.16683 2.062396 0.6070312  0.8842899
 545
## 18         4     3    18 1038.0826 142.75380 1.798205 0.6269541  0.8847202
 654
## 19         5     3    19  713.8594 121.16117 2.073190 0.6047402  0.8793225
 893
## 20         6     3    20  523.0936 101.81040 1.991062 0.6268624  0.8916426
1410
## 21         U     3    21  470.2807  95.31744 1.845806 0.6397602  0.8896560
1497
```

Modeling

For each of the measured variables, a separate linear model was fit, with Trial and Compound ID as predictor variables

```
# Area
area_fit <- lm(Area_m ~ Trial + Comp_ID, data=dat)

# Perimeter
perim_fit <- lm(Perim_m ~ Trial + Comp_ID, data=dat)

# Aspect ratio
ar_fit <- lm(AR_m ~ Trial + Comp_ID, data=dat)

# Circularity
circ_fit <- lm(Circ_m ~ Trial + Comp_ID, data=dat)
```

```r
# Solidity
Solidity_fit <- lm(Solidity_m ~ Trial + Comp_ID, data=dat)
```

Multiple comparisons

Multiple contrast tests are done with respect to the correlations between the dependent variables of the individual models using multiple marginal models.

```r
ni <- as.numeric(table(dat$Comp_ID))
cmt <- contrMat(ni, type="Tukey")

# Multiple marginal models
meandiff <- glht(mmm(area_fit=area_fit,
                     perim_fit=perim_fit,
                     ar_fit=ar_fit,
                     circ_fit=circ_fit,
                     Solidity_fit=Solidity_fit),
                 mlf(mcp(Comp_ID = "Tukey")))

#
meandiff_pval <- fortify(summary(meandiff)) %>%
        mutate(sig=p<0.05) %>%
        dplyr::select(-se, -t) %>%
        data.frame(row.names = NULL)

meandiff_pval

##                     lhs rhs      estimate            p   sig
## 1       area_fit: 2 - 1   0  5.821430e+01 9.567286e-01 FALSE
## 2       area_fit: 3 - 1   0  3.193556e+02 4.022338e-13  TRUE
## 3       area_fit: 4 - 1   0  4.812972e+02 0.000000e+00  TRUE
## 4       area_fit: 5 - 1   0  1.661870e+02 5.624894e-03  TRUE
## 5       area_fit: 6 - 1   0 -4.115868e+01 9.970425e-01 FALSE
## 6       area_fit: U - 1   0 -1.106067e+02 2.625072e-01 FALSE
## 7       area_fit: 3 - 2   0  2.611413e+02 4.043728e-07  TRUE
## 8       area_fit: 4 - 2   0  4.230829e+02 1.409983e-14  TRUE
## 9       area_fit: 5 - 2   0  1.079727e+02 4.786193e-01 FALSE
## 10      area_fit: 6 - 2   0 -9.937298e+01 6.440247e-01 FALSE
## 11      area_fit: U - 2   0 -1.688210e+02 4.168936e-02  TRUE
## 12      area_fit: 4 - 3   0  1.619416e+02 7.112023e-03  TRUE
## 13      area_fit: 5 - 3   0 -1.531686e+02 5.039825e-07  TRUE
## 14      area_fit: 6 - 3   0 -3.605142e+02 0.000000e+00  TRUE
## 15      area_fit: U - 3   0 -4.299623e+02 0.000000e+00  TRUE
## 16      area_fit: 5 - 4   0 -3.151102e+02 1.444178e-12  TRUE
## 17      area_fit: 6 - 4   0 -5.224559e+02 0.000000e+00  TRUE
## 18      area_fit: U - 4   0 -5.919039e+02 0.000000e+00  TRUE
## 19      area_fit: 6 - 5   0 -2.073457e+02 8.782701e-09  TRUE
## 20      area_fit: U - 5   0 -2.767937e+02 4.204970e-12  TRUE
## 21      area_fit: U - 6   0 -6.944802e+01 8.355530e-01 FALSE
## 22     perim_fit: 2 - 1   0  9.372398e+00 8.835281e-01 FALSE
## 23     perim_fit: 3 - 1   0  1.526148e+01 2.544424e-01 FALSE
## 24     perim_fit: 4 - 1   0  2.732035e+01 3.993781e-05  TRUE
## 25     perim_fit: 5 - 1   0  9.258229e+00 8.913982e-01 FALSE
## 26     perim_fit: 6 - 1   0 -1.721450e+01 1.187468e-01 FALSE
## 27     perim_fit: U - 1   0 -2.485152e+01 1.455427e-03  TRUE
```

```
## 28      perim_fit: 3 - 2   0  5.889078e+00 9.993070e-01 FALSE
## 29      perim_fit: 4 - 2   0  1.794796e+01 3.178668e-01 FALSE
## 30      perim_fit: 5 - 2   0 -1.141689e-01 1.000000e+00 FALSE
## 31      perim_fit: 6 - 2   0 -2.658690e+01 2.352845e-02  TRUE
## 32      perim_fit: U - 2   0 -3.422392e+01 3.636029e-04  TRUE
## 33      perim_fit: 4 - 3   0  1.205888e+01 1.428601e-02  TRUE
## 34      perim_fit: 5 - 3   0 -6.003247e+00 9.932483e-01 FALSE
## 35      perim_fit: 6 - 3   0 -3.247598e+01 2.168632e-08  TRUE
## 36      perim_fit: U - 3   0 -4.011300e+01 4.918288e-12  TRUE
## 37      perim_fit: 5 - 4   0 -1.806213e+01 6.855940e-02 FALSE
## 38      perim_fit: 6 - 4   0 -4.453486e+01 1.643130e-14  TRUE
## 39      perim_fit: U - 4   0 -5.217188e+01 0.000000e+00  TRUE
## 40      perim_fit: 6 - 5   0 -2.647273e+01 3.959769e-03  TRUE
## 41      perim_fit: U - 5   0 -3.410975e+01 6.358882e-06  TRUE
## 42      perim_fit: U - 6   0 -7.637018e+00 9.883507e-01 FALSE
## 43         ar_fit: 2 - 1   0 -2.381755e-02 1.000000e+00 FALSE
## 44         ar_fit: 3 - 1   0 -5.693572e-02 9.999872e-01 FALSE
## 45         ar_fit: 4 - 1   0 -1.615734e-01 9.005095e-01 FALSE
## 46         ar_fit: 5 - 1   0  2.655749e-01 2.741016e-01 FALSE
## 47         ar_fit: 6 - 1   0 -1.215077e-01 9.856159e-01 FALSE
## 48         ar_fit: U - 1   0 -1.850910e-01 7.897582e-01 FALSE
## 49         ar_fit: 3 - 2   0 -3.311817e-02 1.000000e+00 FALSE
## 50         ar_fit: 4 - 2   0 -1.377558e-01 9.926260e-01 FALSE
## 51         ar_fit: 5 - 2   0  2.893924e-01 5.159686e-01 FALSE
## 52         ar_fit: 6 - 2   0 -9.769018e-02 9.991979e-01 FALSE
## 53         ar_fit: U - 2   0 -1.612734e-01 6.096741e-01 FALSE
## 54         ar_fit: 4 - 3   0 -1.046377e-01 9.995593e-01 FALSE
## 55         ar_fit: 5 - 3   0  3.225106e-01 4.063017e-01 FALSE
## 56         ar_fit: 6 - 3   0 -6.457201e-02 9.999947e-01 FALSE
## 57         ar_fit: U - 3   0 -1.281552e-01 9.813431e-01 FALSE
## 58         ar_fit: 5 - 4   0  4.271483e-01 9.221319e-02 FALSE
## 59         ar_fit: 6 - 4   0  4.006564e-02 1.000000e+00 FALSE
## 60         ar_fit: U - 4   0 -2.351759e-02 1.000000e+00 FALSE
## 61         ar_fit: 6 - 5   0 -3.870826e-01 1.573353e-01 FALSE
## 62         ar_fit: U - 5   0 -4.506658e-01 2.169425e-02  TRUE
## 63         ar_fit: U - 6   0 -6.358323e-02 9.999716e-01 FALSE
## 64       circ_fit: 2 - 1   0 -1.919853e-02 9.987607e-01 FALSE
## 65       circ_fit: 3 - 1   0  1.000416e-01 1.667472e-04  TRUE
## 66       circ_fit: 4 - 1   0  1.022090e-01 2.988963e-04  TRUE
## 67       circ_fit: 5 - 1   0  5.812449e-02 2.300002e-01 FALSE
## 68       circ_fit: 6 - 1   0  1.273948e-01 5.310850e-08  TRUE
## 69       circ_fit: U - 1   0  1.352451e-01 5.615386e-09  TRUE
## 70       circ_fit: 3 - 2   0  1.192401e-01 3.600350e-08  TRUE
## 71       circ_fit: 4 - 2   0  1.214075e-01 1.079894e-05  TRUE
## 72       circ_fit: 5 - 2   0  7.732302e-02 7.307088e-02 FALSE
## 73       circ_fit: 6 - 2   0  1.465934e-01 1.305033e-10  TRUE
## 74       circ_fit: U - 2   0  1.544436e-01 3.895773e-13  TRUE
## 75       circ_fit: 4 - 3   0  2.167395e-03 1.000000e+00 FALSE
## 76       circ_fit: 5 - 3   0 -4.191709e-02 6.690935e-01 FALSE
## 77       circ_fit: 6 - 3   0  2.735325e-02 8.217284e-01 FALSE
## 78       circ_fit: U - 3   0  3.520354e-02 2.056416e-01 FALSE
## 79       circ_fit: 5 - 4   0 -4.408449e-02 8.120621e-01 FALSE
## 80       circ_fit: 6 - 4   0  2.518586e-02 9.904561e-01 FALSE
## 81       circ_fit: U - 4   0  3.303614e-02 9.060071e-01 FALSE
```

```
## 82      circ_fit: 6 - 5   0  6.927034e-02 8.670402e-02 FALSE
## 83      circ_fit: U - 5   0  7.712063e-02 1.507736e-02  TRUE
## 84      circ_fit: U - 6   0  7.850283e-03 9.999964e-01 FALSE
## 85  Solidity_fit: 2 - 1   0 -1.703145e-02 9.872190e-01 FALSE
## 86  Solidity_fit: 3 - 1   0  8.416879e-02 1.281805e-07  TRUE
## 87  Solidity_fit: 4 - 1   0  8.225511e-02 2.536434e-07  TRUE
## 88  Solidity_fit: 5 - 1   0  7.005196e-02 2.622702e-05  TRUE
## 89  Solidity_fit: 6 - 1   0  9.830650e-02 2.221284e-10  TRUE
## 90  Solidity_fit: U - 1   0  9.764945e-02 3.176529e-10  TRUE
## 91  Solidity_fit: 3 - 2   0  1.012002e-01 0.000000e+00  TRUE
## 92  Solidity_fit: 4 - 2   0  9.928656e-02 1.559530e-12  TRUE
## 93  Solidity_fit: 5 - 2   0  8.708341e-02 6.960377e-11  TRUE
## 94  Solidity_fit: 6 - 2   0  1.153379e-01 0.000000e+00  TRUE
## 95  Solidity_fit: U - 2   0  1.146809e-01 0.000000e+00  TRUE
## 96  Solidity_fit: 4 - 3   0 -1.913676e-03 1.000000e+00 FALSE
## 97  Solidity_fit: 5 - 3   0 -1.411683e-02 7.116117e-01 FALSE
## 98  Solidity_fit: 6 - 3   0  1.413771e-02 1.164414e-02  TRUE
## 99  Solidity_fit: U - 3   0  1.348066e-02 7.182251e-02 FALSE
## 100 Solidity_fit: 5 - 4   0 -1.220315e-02 9.907477e-01 FALSE
## 101 Solidity_fit: 6 - 4   0  1.605139e-02 8.381229e-01 FALSE
## 102 Solidity_fit: U - 4   0  1.539433e-02 8.820907e-01 FALSE
## 103 Solidity_fit: 6 - 5   0  2.825454e-02 1.518039e-02  TRUE
## 104 Solidity_fit: U - 5   0  2.759748e-02 3.003614e-02  TRUE
## 105 Solidity_fit: U - 6   0 -6.570534e-04 1.000000e+00 FALSE
```

Graphical overview about the multiple comparisons

The graphic displays the estimated differences between the different means estimated based on the models shown above $\hat{\mu}_i - \hat{\mu}_{i'}$ (black dots). The error bars indicate 95 % simultaneous confidence intervals for the mean differences. The black line indicates the null hypothesis $H_0\colon \mu_i - \mu_{i'} = 0$. Hence, mean differences do not significantly differ from zero, if their confidence intervals encompass zero.

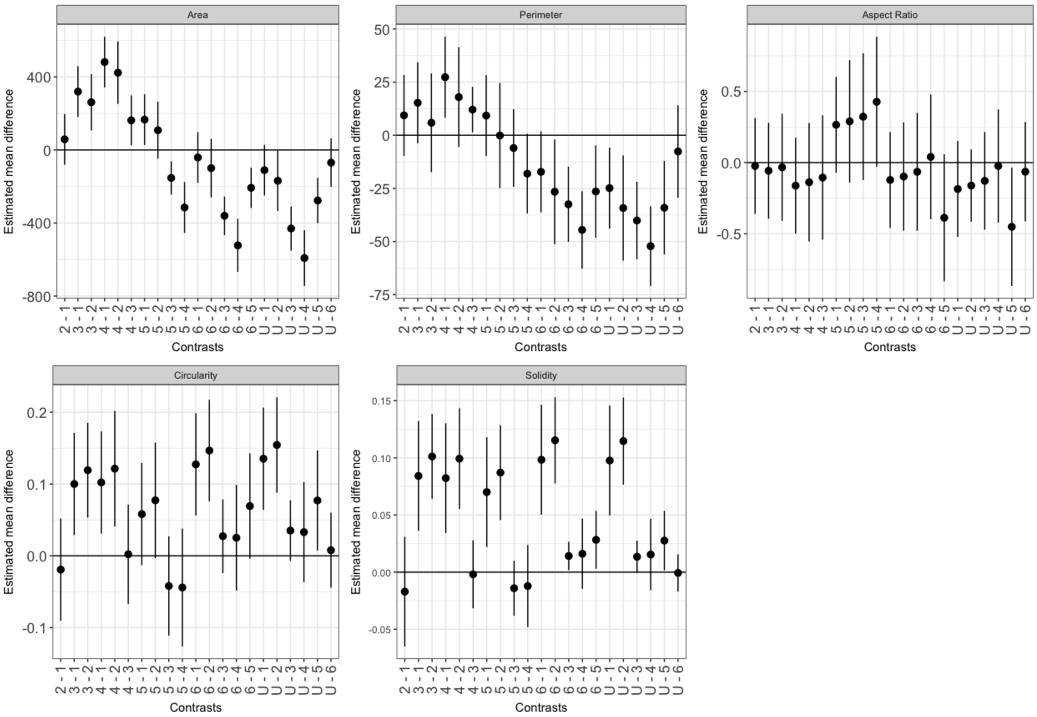

Statistical supplementary statS5: Branches

Bergmann et al. 2024

Packages used for evaluation

```
library(tidyverse)
library(emmeans)
library(lme4)
library(multcomp)
library(tukeytrend)
library(ggpubr)
```

Overview about the data

The complete experiment was run in three separate repetitions (Variable trial). For treatment (six compounds plus untreated control), cells were cultured on one well per treatment within each of the trials. For each well, the number of branches and the number of junctions was counted on several hundreds of cells (variable n) resulting in a total of 16632 analysed cells. Modelling was done based on the average measurement per well.

This data sets (dat) is depicted below and contains the following variables:

- Compound: Compound of interest
- Comp_ID: ID for the compounds of interest
- Trial: Indicator variable for the three repetitions
- Well: Indicator variable for the wells in each repetition
- Branches_m: Average number of branches area per well
- Junctions_m: Average number of junctions per well
- n: Number of cells per well

```
##                   Compound Comp_ID Trial Well Branches_m Junctions_m
n
## 1               pycnidione       1     1    1   1.828418  0.41286863

373
## 2             eupenifeldin       2     1    2   3.035484  1.01290323

310
## 3             xenovulene B       3     1    3   1.304065  0.15121951

615
## 4       hydroxyxenovulene B       4     1    4   1.612245  0.30612245

392
## 5   dehydroxy norpycnidione       5     1    5   1.264010  0.13325031

803
## 6   hydroxy norxenovulene B       6     1    6   1.138355  0.07225211

1301
## 7                untreated       U     1    7   1.105795  0.05727763

1484
## 8               pycnidione       1     2    8   1.997207  0.49441341
```

```
358
## 9              eupenifeldin       2     2    9    2.043860   0.52192982

228
## 10             xenovulene B       3     2   10    1.291667   0.14583333

576
## 11      hydroxyxenovulene B       4     2   11    1.196746   0.09763314

676
## 12 dehydroxy norpycnidione       5     2   12    1.248196   0.12409812

693
## 13 hydroxy norxenovulene B       6     2   13    1.113966   0.05928854

1518
## 14                untreated      U     2   14    1.120135   0.06373378

1773
## 15               pycnidione      1     3   15    1.891753   0.44329897

194
## 16              eupenifeldin     2     3   16    2.029499   0.51032448

339
## 17             xenovulene B      3     3   17    1.255046   0.12660550

545
## 18      hydroxyxenovulene B      4     3   18    1.238532   0.12079511

654
## 19 dehydroxy norpycnidione       5     3   19    1.178052   0.09182531

893
## 20 hydroxy norxenovulene B       6     3   20    1.097872   0.05319149

1410
## 21                untreated      U     3   21    1.122244   0.06680027

1497
```

Modeling

For each of the measured variables, a separate linear model was fit, with Trial and Compound ID as predictor variables

```
# Area
branches_fit <- lm(Branches_m ~ Trial + Comp_ID, data=dat)

# Perimeter
junctions_fit <- lm(Junctions_m ~ Trial + Comp_ID, data=dat)
```

Multiple comparisons

Multiple contrast tests are done with respect to the correlations between the dependent variables of the individual models using multiple marginal models.

```r
ni <- as.numeric(table(dat$Comp_ID))
cmt <- contrMat(ni, type="Tukey")

# Multiple marginal models
meandiff <- glht(mmm(branches_fit=branches_fit,
                     junctions_fit=junctions_fit),
                 mlf(mcp(Comp_ID = "Tukey")))

#
meandiff_pval <- fortify(summary(meandiff)) %>%
        mutate(sig=p<0.05) %>%
        dplyr::select(-se, -t) %>%
        data.frame(row.names = NULL)

meandiff_pval

##                      lhs rhs      estimate            p   sig
## 1   branches_fit: 2 - 1   0  0.4638215111 1.428023e-01 FALSE
## 2   branches_fit: 3 - 1   0 -0.6221999776 1.122805e-02  TRUE
## 3   branches_fit: 4 - 1   0 -0.5566183139 3.676095e-02  TRUE
## 4   branches_fit: 5 - 1   0 -0.6757065964 4.376842e-03  TRUE
## 5   branches_fit: 6 - 1   0 -0.7890614385 3.379284e-04  TRUE
## 6   branches_fit: U - 1   0 -0.7897341703 3.398045e-04  TRUE
## 7   branches_fit: 3 - 2   0 -1.0860214888 2.849172e-06  TRUE
## 8   branches_fit: 4 - 2   0 -1.0204398250 1.139823e-04  TRUE
## 9   branches_fit: 5 - 2   0 -1.1395281075 7.583477e-07  TRUE
## 10  branches_fit: 6 - 2   0 -1.2528829496 5.874713e-08  TRUE
## 11  branches_fit: U - 2   0 -1.2535556814 4.073595e-08  TRUE
## 12  branches_fit: 4 - 3   0  0.0655816638 9.988450e-01 FALSE
## 13  branches_fit: 5 - 3   0 -0.0535066188 9.992345e-01 FALSE
## 14  branches_fit: 6 - 3   0 -0.1668614609 7.929603e-01 FALSE
## 15  branches_fit: U - 3   0 -0.1675341926 8.639582e-01 FALSE
## 16  branches_fit: 5 - 4   0 -0.1190882825 9.651053e-01 FALSE
## 17  branches_fit: 6 - 4   0 -0.2324431246 5.490846e-01 FALSE
## 18  branches_fit: U - 4   0 -0.2331158564 6.490952e-01 FALSE
## 19  branches_fit: 6 - 5   0 -0.1133548421 9.538080e-01 FALSE
## 20  branches_fit: U - 5   0 -0.1140275739 9.737858e-01 FALSE
## 21  branches_fit: U - 6   0 -0.0006727318 1.000000e+00 FALSE
## 22 junctions_fit: 2 - 1   0  0.2315255082 1.387972e-01 FALSE
## 23 junctions_fit: 3 - 1   0 -0.3089742198 1.162076e-02  TRUE
## 24 junctions_fit: 4 - 1   0 -0.2753434392 3.862750e-02  TRUE
## 25 junctions_fit: 5 - 1   0 -0.3338024221 4.386203e-03  TRUE
## 26 junctions_fit: 6 - 1   0 -0.3886162896 2.324123e-04  TRUE
## 27 junctions_fit: U - 1   0 -0.3875897766 3.191971e-04  TRUE
## 28 junctions_fit: 3 - 2   0 -0.5404997280 3.152466e-06  TRUE
## 29 junctions_fit: 4 - 2   0 -0.5068689473 2.144680e-05  TRUE
## 30 junctions_fit: 5 - 2   0 -0.5653279303 7.437242e-07  TRUE
## 31 junctions_fit: 6 - 2   0 -0.6201417978 3.168100e-08  TRUE
## 32 junctions_fit: U - 2   0 -0.6191152848 6.963035e-08  TRUE
## 33 junctions_fit: 4 - 3   0  0.0336307807 9.986919e-01 FALSE
## 34 junctions_fit: 5 - 3   0 -0.0248282022 9.995081e-01 FALSE
## 35 junctions_fit: 6 - 3   0 -0.0796420698 8.313510e-01 FALSE
## 36 junctions_fit: U - 3   0 -0.0786155568 8.985300e-01 FALSE
## 37 junctions_fit: 5 - 4   0 -0.0584589829 9.689262e-01 FALSE
```

```
## 38 junctions_fit: 6 - 4   0 -0.1132728505 5.877227e-01 FALSE
## 39 junctions_fit: U - 4   0 -0.1122463374 6.955207e-01 FALSE
## 40 junctions_fit: 6 - 5   0 -0.0548138676 9.618961e-01 FALSE
## 41 junctions_fit: U - 5   0 -0.0537873545 9.810913e-01 FALSE
## 42 junctions_fit: U - 6   0  0.0010265130 1.000000e+00 FALSE
```

Graphical overview about the multiple comparisons

The graphic displays the estimated differences between the different means estimated based on the models shown above $\hat{\mu}_i - \hat{\mu}_{i'}$ (black dots). The error bars indicate 95 % simultaneous confidence intervals for the mean differences. The black line indicates the null hypothesis $H_0: \mu_i - \mu_{i'} = 0$. Hence, mean differences do not significantly differ from zero, if their confidence intervals encompass zero.

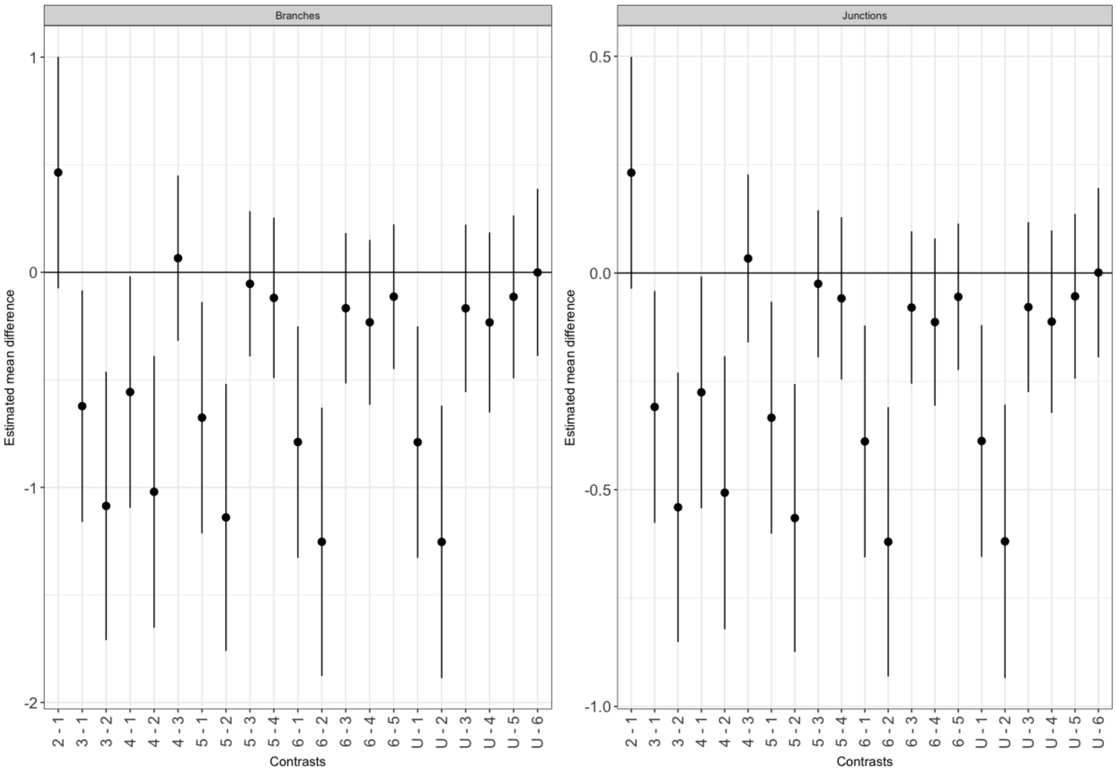

Statistical supplementary statS6: Proliferation for 4-hydroxy norxenovulene B 6 and DMSO

Bergmann et al. 2024

```
# Load the packages used for evaluation
library(lme4)
library(lmerTest)
library(tidyverse)
library(emmeans)
library(multcomp)
```

Data set

- Treatment: Name of the treatments (combination of compound and dose)
- Rep: Repetition (timepoint)
- Plate: Plate per timepoint
- Prop: Proportion of the fluorescence signals at the start of the experiment and its end
- Fluo1: Fluorescence signals at the start of the experiments (day one)
- Fluo3: Fluorescence signals at the end of the experiments (day three)

```
proli7

##                          Treatment Rep Plate      Prop Fluo1 Fluo3
## 1                        untreated   1     1 12.220345 33276  2723
## 2   4-hydroxy norxenovulene B 6:4   1     1 12.148959 33276  2739
## 3   4-hydroxy norxenovulene B 6:6   1     1 11.867332 33276  2804
## 4   4-hydroxy norxenovulene B 6:8   1     1 12.052155 33276  2761
## 5  4-hydroxy norxenovulene B 6:12   1     1 11.679888 33276  2849
## 6                        untreated   1     2 12.338154 33276  2697
## 7                         DMSO:0.4   1     2 12.338154 33276  2697
## 8                         DMSO:0.6   1     2 12.078403 33276  2755
## 9                         DMSO:0.8   1     2 12.434978 33276  2676
## 10                        DMSO:1.2   1     2 11.522161 33276  2888
## 11                       untreated   2     3  9.097393 36990  4066
## 12  4-hydroxy norxenovulene B 6:4   2     3  9.153675 36990  4041
## 13  4-hydroxy norxenovulene B 6:6   2     3  8.838710 36990  4185
## 14  4-hydroxy norxenovulene B 6:8   2     3  8.784137 36990  4211
## 15 4-hydroxy norxenovulene B 6:12   2     3  8.534841 36990  4334
## 16                       untreated   2     4  9.097393 36990  4066
## 17                        DMSO:0.4   2     4  9.286970 36990  3983
## 18                        DMSO:0.6   2     4  9.249812 36990  3999
## 19                        DMSO:0.8   2     4  9.097393 36990  4066
## 20                        DMSO:1.2   2     4  8.586351 36990  4308
## 21                       untreated   3     5 11.146008 42444  3808
## 22  4-hydroxy norxenovulene B 6:4   3     5 11.102276 42444  3823
## 23  4-hydroxy norxenovulene B 6:6   3     5 10.967442 42444  3870
## 24  4-hydroxy norxenovulene B 6:8   3     5 10.154067 42444  4180
## 25 4-hydroxy norxenovulene B 6:12   3     5  9.977433 42444  4254
## 26                       untreated   3     6 11.146008 42444  3808
## 27                        DMSO:0.4   3     6 10.699269 42444  3967
## 28                        DMSO:0.6   3     6 10.767123 42444  3942
## 29                        DMSO:0.8   3     6 10.354721 42444  4099
## 30                        DMSO:1.2   3     6  9.872994 42444  4299
```

Statistical model

- Linear mixed effects model
- Fluorescence proportion as response variable (ln-transformed)
- Repetition and Treatment as fixed effects
- Plates nested in Repetition as normal distributed random effects

```
fit_p7 <- lmer(log(Prop) ~ Rep + Treatment  + (1|Rep:Plate), data=proli7)
```

Model based means and their pointwise 95% confidence intervals

- All mean comparisons were performed on the ln-scale
- The means and their CI are back transformed from the ln-scale to the response scale

```
means <- emmeans(fit_p7,
                 specs="Treatment",
                 type="response")

means

##                          Treatment  response         SE  lower.CL  upper.CL
## 1                        untreated 10.758070 0.09157412 10.566103 10.95353
## 2  4-hydroxy norxenovulene B 6:4 10.728754 0.13274648 10.452111 11.01272
## 3  4-hydroxy norxenovulene B 6:6 10.478912 0.12965520 10.208712 10.75626
## 4  4-hydroxy norxenovulene B 6:8 10.244758 0.12675801  9.980595 10.51591
## 5 4-hydroxy norxenovulene B 6:12  9.982778 0.12351654  9.725370 10.24700
## 6                         DMSO:0.4 10.701854 0.13241364 10.425904 10.98511
## 7                         DMSO:0.6 10.634415 0.13157923 10.360205 10.91588
## 8                         DMSO:0.8 10.540625 0.13041876 10.268833 10.81961
## 9                         DMSO:1.2  9.921181 0.12275441  9.665362 10.18377
```

A graphical overview about this table is given in the supplementary figure S 5 c.

Mean comparisons (contrast tests)

Pairwise mean comparisons, that are defined below by `cont_list`, were run based on mean differences $ln(\hat{\mu}_j) - ln(\hat{\mu}_{j'})$. The tested null-hypothesis for each comparison was $H_0: ln(\mu_j) - ln(\mu_{j'}) = 0$. The table below shows results from the tests that are already back transformed to the response scale. Hence the back transformed contrasts become proportions on the response scale $exp\left(ln(\hat{\mu}_j) - ln(\hat{\mu}_{j'})\right) = \hat{\mu}_j/\hat{\mu}_{j'}$. Similarly, the H_0 for the back transformed contrasts changes to $H_0: \mu_j/\mu_{j'} = 1$

```
# List of contrast coefficients
cont_list

## $`4-hydroxy norxenovulene B 6:4 - untreated`
## [1] -1  1  0  0  0  0  0  0  0
##
## $`4-hydroxy norxenovulene B 6:6 - untreated`
## [1] -1  0  1  0  0  0  0  0  0
##
## $`4-hydroxy norxenovulene B 6:8 - untreated`
## [1] -1  0  0  1  0  0  0  0  0
##
## $`4-hydroxy norxenovulene B 6:12 - untreated`
```

```
## [1] -1  0  0  0  1  0  0  0  0
##
## $`DMSO:0.4 - untreated`
## [1] -1  0  0  0  0  1  0  0  0
##
## $`DMSO:0.6 - untreated`
## [1] -1  0  0  0  0  0  1  0  0
##
## $`DMSO:0.8 - untreated`
## [1] -1  0  0  0  0  0  0  1  0
##
## $`DMSO:1.2 - untreated`
## [1] -1  0  0  0  0  0  0  0  1
##
## $`DMSO:0.4 - 4-hydroxy norxenovulene B 6:4`
## [1]  0 -1  0  0  0  1  0  0  0
##
## $`DMSO:0.6 - 4-hydroxy norxenovulene B 6:6`
## [1]  0  0 -1  0  0  0  1  0  0
##
## $`DMSO:0.8 - 4-hydroxy norxenovulene B 6:8`
## [1]  0  0  0 -1  0  0  0  1  0
##
## $`DMSO:1.2 - 4-hydroxy norxenovulene B 6:12`
## [1]  0  0  0  0 -1  0  0  0  1
```

```
# Multiple contrast tests
comp <- contrast(means,
                 method=cont_list,
                 adjust="mvt")
```

```
##                                       contrast     ratio p.value   sig
## 1  4-hydroxy norxenovulene B 6:12 / untreated 0.9279339   0.001  TRUE
## 2   4-hydroxy norxenovulene B 6:4 / untreated 0.9972749  >0.999 FALSE
## 3   4-hydroxy norxenovulene B 6:6 / untreated 0.9740513   0.522 FALSE
## 4   4-hydroxy norxenovulene B 6:8 / untreated 0.9522858   0.036  TRUE
## 5    DMSO:0.4 / 4-hydroxy norxenovulene B 6:4 0.9974927  >0.999 FALSE
## 6                         DMSO:0.4 / untreated 0.9947745  >0.999 FALSE
## 7    DMSO:0.6 / 4-hydroxy norxenovulene B 6:6 1.0148396   0.981 FALSE
## 8                         DMSO:0.6 / untreated 0.9885058   0.986 FALSE
## 9    DMSO:0.8 / 4-hydroxy norxenovulene B 6:8 1.0288798   0.648 FALSE
## 10                        DMSO:0.8 / untreated 0.9797877   0.775 FALSE
## 11  DMSO:1.2 / 4-hydroxy norxenovulene B 6:12 0.9938297  >0.999 FALSE
## 12                        DMSO:1.2 / untreated 0.9222082  <0.001  TRUE
```

The contrast ratios, their corresponding p-values and their confidence intervals (table below) are shown in supplementary figure S 5 d.

```
confint(comp)
```

```
##  contrast                                   ratio     SE   df lower.CL u
pper.CL
##  4-hydroxy norxenovulene B 6:4 / untreated  0.997 0.0147 18.6    0.952
  1.045
##  4-hydroxy norxenovulene B 6:6 / untreated  0.974 0.0144 18.6    0.930
```

```
  1.020
##  4-hydroxy norxenovulene B 6:8 / untreated  0.952 0.0141 18.6    0.909
  0.998
##  4-hydroxy norxenovulene B 6:12 / untreated 0.928 0.0137 18.6    0.886
  0.972
##  DMSO:0.4 / untreated                       0.995 0.0147 18.6    0.950
  1.042
##  DMSO:0.6 / untreated                       0.989 0.0146 18.6    0.944
  1.036
##  DMSO:0.8 / untreated                       0.980 0.0145 18.6    0.935
  1.026
##  DMSO:1.2 / untreated                       0.922 0.0136 18.6    0.880
  0.966
##  DMSO:0.4 / 4-hydroxy norxenovulene B 6:4   0.997 0.0179 17.2    0.942
  1.056
##  DMSO:0.6 / 4-hydroxy norxenovulene B 6:6   1.015 0.0182 17.2    0.959
  1.074
##  DMSO:0.8 / 4-hydroxy norxenovulene B 6:8   1.029 0.0185 17.2    0.972
  1.089
##  DMSO:1.2 / 4-hydroxy norxenovulene B 6:12  0.994 0.0178 17.2    0.939
  1.052
##
## Results are averaged over the levels of: Rep
## Degrees-of-freedom method: kenward-roger
## Confidence level used: 0.95
## Conf-level adjustment: mvt method for 12 estimates
## Intervals are back-transformed from the log scale
```

Statistical supplementary statS7: Proliferation

Bergmann et al. 2024

```
# Load the packages used for evaluation
library(tidyverse)
library(emmeans)
library(multcomp)
```

Data set

- Compound: Name of the treatments
- Rep: Repetition (timepoint)
- Prop: Proportions of the fluorescence signals at the start of the experiment and its end
- Fluo1: Fluorescence signals at the start of the experiments (day one)
- Fluo3: Fluorescence signals at the end of the experiments (day three)

```
proli

##                         Compound Rep      Prop Fluo1 Fluo3
## 1                   xenovulene B 3   1  5.611080 59758 10650
## 2                   eupenifeldin 2   1  5.509681 59758 10846
## 3  4-dehydroxy norpycnidione 5   1  5.765920 59758 10364
## 4       4-hydroxyxenovulene B 4   1  5.857479 59758 10202
## 5                   pycnidione 1   1  5.870714 59758 10179
## 6  4-hydroxy norxenovulene B 6   1 12.898338 59758  4633
## 7                      untreated   1 14.221323 59758  4202
## 8                   xenovulene B 3   2  6.730180 64603  9599
## 9                   eupenifeldin 2   2  6.730180 64603  9599
## 10 4-dehydroxy norpycnidione 5   2  6.346694 64603 10179
## 11      4-hydroxyxenovulene B 4   2  7.140820 64603  9047
## 12                  pycnidione 1   2  6.899082 64603  9364
## 13 4-hydroxy norxenovulene B 6   2 14.968258 64603  4316
## 14                     untreated   2 15.888588 64603  4066
## 15                  xenovulene B 3   3  6.417296 50164  7817
## 16                  eupenifeldin 2   3  9.086035 50164  5521
## 17 4-dehydroxy norpycnidione 5   3  6.590121 50164  7612
## 18      4-hydroxyxenovulene B 4   3  6.796369 50164  7381
## 19                  pycnidione 1   3  7.598304 50164  6602
## 20 4-hydroxy norxenovulene B 6   3 15.178215 50164  3305
## 21                     untreated   3 15.607965 50164  3214
```

Linear model

The data can be treated as a block-design with replicates as blocks and compund as variable of interest.

- Rep as blocks
- Compund as treatments
- Prop (ln-transformed proportions) as dependent variable

```
fit <- lm(log(Prop) ~ Rep + Compound, data=proli)
```

The fitted model is $ln(y_{ij}) = \hat{\mu} + \hat{r}_i + \hat{c}_j + \hat{e}_{ij}$ with:

- $ln(y_{ij})$: Ln-transformed proportions (signal on day 1 / signal on day 3)
- $\hat{\mu}$: Fitted overall mean proportion
- $\hat{r}_i$: Observed effects due to the replication, with $i = 1,2$
- $\hat{c}_j$: Observed effects of the compounds, with $j = 1, \dots, 7$
- $\hat{e}_{ij}$: Observed residuals

Analysis of variance

```
anova(fit)

## Analysis of Variance Table
##
## Response: log(Prop)
##           Df  Sum Sq Mean Sq F value    Pr(>F)
## Rep        2 0.16006 0.08003  13.226 0.0009237 ***
## Compound   6 2.87086 0.47848  79.078 6.044e-09 ***
## Residuals 12 0.07261 0.00605
## ---
## Signif. codes:  0 '***' 0.001 '**' 0.01 '*' 0.05 '.' 0.1 ' ' 1
```

- The compound is significant ($\alpha = 0.05 > p = 6.044e - 09$)

Model based least square means and their pointwise 95% confidence intervals

- All mean comparisons were performed on the ln-scale
- The means and their CI are back transformed from the ln-scale to the response scale

```
means <- emmeans(fit,
                 specs="Compound",
                 type="response")

##                     Compound  response        SE df  lower.CL  upper.CL
## 1                  untreated 15.221491 0.6835946 12 13.802617 16.786222
## 2               pycnidione 1  6.751490 0.3032083 12  6.122148  7.445526
## 3             eupenifeldin 2  6.958397 0.3125004 12  6.309768  7.673702
## 4             xenovulene B 3  6.234597 0.2799947 12  5.653438  6.875498
## 5     4-hydroxyxenovulene B 4  6.575245 0.2952931 12  5.962332  7.251163
## 6 4-dehydroxy norpycnidione 5  6.224482 0.2795404 12  5.644266  6.864343
## 7 4-hydroxy norxenovulene B 6 14.310075 0.6426630 12 12.976158 15.781114
```

A graphical overview about this table is given in the manuscript in figure 4 a.

Mean comparisons (contrast tests)

Pairwise mean comparisons were run based on mean differences $ln(\hat{\mu}_j) - ln(\hat{\mu}_{j'})$. The tested null-hypothesis for each comparison was $H_0: ln(\mu_j) - ln(\mu_{j'}) = 0$. The table below shows results from the tests that are already back transformed to the response scale. Hence, the back transformed contrasts become proportions on the response scale

$exp\left(ln(\hat{\mu}_j) - ln(\hat{\mu}_{j'})\right) = \hat{\mu}_j/\hat{\mu}_{j'}$. Similarly, the H_0 for the back transformed contrasts changes to $H_0: \mu_j/\mu_{j'} = 1$

```
# Contrasts are given on the response scale
comp <- contrast(means,
                 method="pairwise",
                 adjust="mvt")

##                                                             contrast ratio p
.value
## 1                                          untreated / pycnidione 1  2.25
<0.001
## 2                                        untreated / eupenifeldin 2  2.19
<0.001
## 3                                        untreated / xenovulene B 3  2.44
<0.001
## 4                             untreated / (4-hydroxyxenovulene B 4)  2.31
<0.001
## 5                         untreated / (4-dehydroxy norpycnidione 5)  2.45
<0.001
## 6                         untreated / (4-hydroxy norxenovulene B 6)  1.06
 0.951
## 7                                   pycnidione 1 / eupenifeldin 2  0.97
 0.999
## 8                                   pycnidione 1 / xenovulene B 3  1.08
  0.86
## 9                          pycnidione 1 / (4-hydroxyxenovulene B 4)  1.03
 0.999
## 10                     pycnidione 1 / (4-dehydroxy norpycnidione 5)  1.08
 0.849
## 11                     pycnidione 1 / (4-hydroxy norxenovulene B 6)  0.47
<0.001
## 12                                eupenifeldin 2 / xenovulene B 3  1.12
 0.612
## 13                       eupenifeldin 2 / (4-hydroxyxenovulene B 4)  1.06
 0.967
## 14                   eupenifeldin 2 / (4-dehydroxy norpycnidione 5)  1.12
 0.597
## 15                   eupenifeldin 2 / (4-hydroxy norxenovulene B 6)  0.49
<0.001
## 16                       xenovulene B 3 / (4-hydroxyxenovulene B 4)  0.95
 0.976
## 17                   xenovulene B 3 / (4-dehydroxy norpycnidione 5)  1.00
>0.999
## 18                   xenovulene B 3 / (4-hydroxy norxenovulene B 6)  0.44
<0.001
## 19    (4-hydroxyxenovulene B 4) / (4-dehydroxy norpycnidione 5)  1.06
 0.972
## 20    (4-hydroxyxenovulene B 4) / (4-hydroxy norxenovulene B 6)  0.46
<0.001
## 21 (4-dehydroxy norpycnidione 5) / (4-hydroxy norxenovulene B 6)  0.43
<0.001
```

Results of the mean comparisons are summarized in the manuscript in figure 4 a. The contrast ratios, their corresponding p-values and their confidence intervals (table below) are shown in figure 4 b in the manuscript.

```
# 95% simultanious confidence intervals for the contrasts on response scale
confint(contrast(means,
                 method="pairwise",
                 adjust="mvt"))

##                                                         contrast  LCL  U
CL
## 1                                       untreated / pycnidione 1 1.81 2.
82
## 2                                     untreated / eupenifeldin 2 1.75 2.
73
## 3                                     untreated / xenovulene B 3 1.95 3.
05
## 4                          untreated / (4-hydroxyxenovulene B 4) 1.85 2.
89
## 5                      untreated / (4-dehydroxy norpycnidione 5) 1.96 3.
05
## 6                      untreated / (4-hydroxy norxenovulene B 6) 0.85 1.
33
## 7                                  pycnidione 1 / eupenifeldin 2 0.78 1.
21
## 8                                  pycnidione 1 / xenovulene B 3 0.87 1.
35
## 9                       pycnidione 1 / (4-hydroxyxenovulene B 4) 0.82 1.
28
## 10                  pycnidione 1 / (4-dehydroxy norpycnidione 5) 0.87 1.
35
## 11                  pycnidione 1 / (4-hydroxy norxenovulene B 6) 0.38 0.
59
## 12                               eupenifeldin 2 / xenovulene B 3 0.89 1.
39
## 13                    eupenifeldin 2 / (4-hydroxyxenovulene B 4) 0.85 1.
32
## 14                eupenifeldin 2 / (4-dehydroxy norpycnidione 5) 0.90 1.
40
## 15                eupenifeldin 2 / (4-hydroxy norxenovulene B 6) 0.39 0.
61
## 16                    xenovulene B 3 / (4-hydroxyxenovulene B 4) 0.76 1.
18
## 17                xenovulene B 3 / (4-dehydroxy norpycnidione 5) 0.80 1.
25
## 18                xenovulene B 3 / (4-hydroxy norxenovulene B 6) 0.35 0.
54
## 19     (4-hydroxyxenovulene B 4) / (4-dehydroxy norpycnidione 5) 0.85 1.
32
## 20     (4-hydroxyxenovulene B 4) / (4-hydroxy norxenovulene B 6) 0.37 0.
57
## 21 (4-dehydroxy norpycnidione 5) / (4-hydroxy norxenovulene B 6) 0.35 0.
54
```

Statistical supplementary statS8: Toxicity for 4-hydroxy norxenovulene B 6 and DMSO

Bergmann et al 2024

```r
# Load the packages used for evaluation
library(tidyverse)
library(lme4)
library(lmerTest)
library(emmeans)
library(multcomp)
library(multcompView)
```

Data set

- Treatment: Name of the treatments (combination compound and dose)
- Rep: Repetition (timepoint)
- Plate: Plate per timepoint
- Prop: Proportion of the dead cells
- Dead: Number of dead cells,
- Alive: Number of living cells
- Total: Total number of cells

```
##                           Treatment Rep Plate Alive        Prop Total Dead
## 1                         untreated   1     1  9968 0.001502554  9983   15
## 2   4-hydroxy norxenovulene B 6:4   1     1  9295 0.069662696  9991  696
## 3   4-hydroxy norxenovulene B 6:6   1     1  9840 0.014916408  9989  149
## 4   4-hydroxy norxenovulene B 6:8   1     1  9627 0.036046861  9987  360
## 5  4-hydroxy norxenovulene B 6:12   1     1  9422 0.055627944  9977  555
## 6                         untreated   1     2  9973 0.001801621  9991   18
## 7                          DMSO:0.4   1     2  9760 0.023023023  9990  230
## 8                          DMSO:0.6   1     2  9161 0.082891180  9989  828
## 9                          DMSO:0.8   1     2  9761 0.021649795  9977  216
## 10                         DMSO:1.2   1     2  9739 0.024441551  9983  244
## 11                        untreated   2     3  9558 0.011275473  9667  109
## 12  4-hydroxy norxenovulene B 6:4   2     3  9379 0.012216956  9495  116
## 13  4-hydroxy norxenovulene B 6:6   2     3  9097 0.017920760  9263  166
## 14  4-hydroxy norxenovulene B 6:8   2     3  8968 0.011245865  9070  102
## 15 4-hydroxy norxenovulene B 6:12   2     3  8547 0.017699115  8701  154
## 16                        untreated   2     4  9374 0.001916525  9392   18
## 17                         DMSO:0.4   2     4  9313 0.013766811  9443  130
## 18                         DMSO:0.6   2     4  9153 0.012940796  9273  120
## 19                         DMSO:0.8   2     4  9448 0.010058676  9544   96
## 20                         DMSO:1.2   2     4  9051 0.022147796  9256  205
## 21                        untreated   3     5  9569 0.002709745  9595   26
## 22  4-hydroxy norxenovulene B 6:4   3     5  9107 0.040863612  9495  388
## 23  4-hydroxy norxenovulene B 6:6   3     5  9105 0.025473617  9343  238
## 24  4-hydroxy norxenovulene B 6:8   3     5  9425 0.002962023  9453   28
## 25 4-hydroxy norxenovulene B 6:12   3     5  8907 0.033318863  9214  307
## 26                        untreated   3     6  9504 0.003460208  9537   33
## 27                         DMSO:0.4   3     6  9327 0.024270321  9559  232
## 28                         DMSO:0.6   3     6  9261 0.033903609  9586  325
## 29                         DMSO:0.8   3     6  9306 0.015446466  9452  146
## 30                         DMSO:1.2   3     6  8899 0.048540575  9353  454
```

Statistical model

- Generalized linear mixed model
- Avereage proportions of dead cells $\hat{\pi}_j$ are modelled
- Binomial assumption (overdispersed binomial is impossible in `glmer()`)
- Modelling of the response variable was done on logit link $ln\left(\frac{\hat{\pi}_j}{1-\hat{\pi}_j}\right)$
- Repetition and Treatment as fixed effects
- Plates nested in Repetition as random effects

```
fit_t7 <- glmer(cbind(Dead, Alive) ~ Rep + Treatment + (1|Rep:Plate),
                family="binomial",
                data=tox7_num)
```

Model based means and their pointwise 95% confidence intervals

- Average proportions of des cells $\hat{\pi}_j$

```
means <- emmeans(fit_t7,
                 specs="Treatment",
                 type="response")

##                         Treatment   prob     SE asymp.LCL asymp.UCL
## 1                       untreated 0.0033 0.0005    0.0025    0.0044
## 4  4-hydroxy norxenovulene B 6:8 0.0119 0.0018    0.0088    0.0160
## 3  4-hydroxy norxenovulene B 6:6 0.0134 0.0020    0.0100    0.0180
## 8                        DMSO:0.8 0.0194 0.0029    0.0144    0.0260
## 6                        DMSO:0.4 0.0249 0.0037    0.0186    0.0334
## 5 4-hydroxy norxenovulene B 6:12 0.0252 0.0037    0.0189    0.0336
## 2  4-hydroxy norxenovulene B 6:4 0.0291 0.0042    0.0219    0.0387
## 9                        DMSO:1.2 0.0384 0.0056    0.0289    0.0510
## 7                        DMSO:0.6 0.0536 0.0076    0.0405    0.0706
```

A graphical overview about this table is given in the manuscript in supplementary figure S 6 c.

Multiple comparisons

Pairwise mean comparisons, that are defined below by `cont_list`, were run based on log odds-ratios $ln\left(\frac{\hat{\pi}_j}{1-\hat{\pi}_j} / \frac{\hat{\pi}_{j'}}{1-\hat{\pi}_{j'}}\right) = ln\left(\frac{\hat{\pi}_j}{1-\hat{\pi}_j}\right) - ln\left(\frac{\hat{\pi}_{j'}}{1-\hat{\pi}_{j'}}\right)$. The tested null-hypothesis for each comparison was H_0: $ln\left(\frac{\pi_j}{1-\pi_j}\right) - ln\left(\frac{\pi_{j'}}{1-\pi_{j'}}\right) = 0$.

```
# List of contrast coefficients
cont_list

## $`4-hydroxy norxenovulene B 6:4 - untreated`
## [1] -1  1  0  0  0  0  0  0  0
##
## $`4-hydroxy norxenovulene B 6:6 - untreated`
## [1] -1  0  1  0  0  0  0  0  0
##
## $`4-hydroxy norxenovulene B 6:8 - untreated`
## [1] -1  0  0  1  0  0  0  0  0
```

```
##
## $`4-hydroxy norxenovulene B 6:12 - untreated`
## [1] -1  0  0  0  1  0  0  0  0
##
## $`DMSO:0.4 - untreated`
## [1] -1  0  0  0  0  1  0  0  0
##
## $`DMSO:0.6 - untreated`
## [1] -1  0  0  0  0  0  1  0  0
##
## $`DMSO:0.8 - untreated`
## [1] -1  0  0  0  0  0  0  1  0
##
## $`DMSO:1.2 - untreated`
## [1] -1  0  0  0  0  0  0  0  1
##
## $`DMSO:0.4 - 4-hydroxy norxenovulene B 6:4`
## [1]  0 -1  0  0  0  1  0  0  0
##
## $`DMSO:0.6 - 4-hydroxy norxenovulene B 6:6`
## [1]  0  0 -1  0  0  0  1  0  0
##
## $`DMSO:0.8 - 4-hydroxy norxenovulene B 6:8`
## [1]  0  0  0 -1  0  0  0  1  0
##
## $`DMSO:1.2 - 4-hydroxy norxenovulene B 6:12`
## [1]  0  0  0  0 -1  0  0  0  1
```

The table below shows results from the tests that are back transformed to the response scale. Hence the back transformed contrasts become proportions on the response scale $exp\left[ln\left(\frac{\hat{\pi}_j}{1-\hat{\pi}_j}\right) - ln\left(\frac{\hat{\pi}_{j'}}{1-\hat{\pi}_{j'}}\right)\right] = \frac{\hat{\pi}_j}{1-\hat{\pi}_j} / \frac{\hat{\pi}_{j'}}{1-\hat{\pi}_{j'}}$. Similarly, the H_0 for the back transformed contrasts changes to $H_0\colon \frac{\pi_{j'}}{1-\pi_{j'}} / \frac{\pi_{j'}}{1-\pi_{j'}} = 1$

```
# Multiple contrast tests
comp <- contrast(means,
                 method=cont_list,
                 adjust="mvt")
```

```
##                                        contrast odds.ratio p.value   sig
## 1  4-hydroxy norxenovulene B 6:12 / untreated  7.7557037  <0.001  TRUE
## 2   4-hydroxy norxenovulene B 6:4 / untreated  8.9839760  <0.001  TRUE
## 3   4-hydroxy norxenovulene B 6:6 / untreated  4.0712481  <0.001  TRUE
## 4   4-hydroxy norxenovulene B 6:8 / untreated  3.6005985  <0.001  TRUE
## 5    DMSO:0.4 / 4-hydroxy norxenovulene B 6:4  0.8528535   0.819 FALSE
## 6                        DMSO:0.4 / untreated  7.6620153  <0.001  TRUE
## 7    DMSO:0.6 / 4-hydroxy norxenovulene B 6:6  4.1679982  <0.001  TRUE
## 8                        DMSO:0.6 / untreated 16.9689545  <0.001  TRUE
## 9    DMSO:0.8 / 4-hydroxy norxenovulene B 6:8  1.6423417    0.03  TRUE
## 10                       DMSO:0.8 / untreated  5.9134131  <0.001  TRUE
## 11  DMSO:1.2 / 4-hydroxy norxenovulene B 6:12  1.5441243    0.06 FALSE
## 12                       DMSO:1.2 / untreated 11.9757703  <0.001  TRUE
```

A graphical overview about this table is given in the manuscript in supplementary figure S 6 d.

Statistical supplementary statS9: Toxicity

Bergmann et al. 2024

```
# Load the packages used for evaluation
library(tidyverse)
library(emmeans)
```

Data set

- Compound: Name of the treatments
- Rep: Repetition (timepoint)
- Total: Total number of cells
- Dead: Number of dead cells
- Prop: Proportion of dead cells (for plotting only)

```
toxy

##    Rep                     Compound Total Dead Alive        Prop
## 1    1               xenovulene B 3  9520  963  8557 0.101155462
## 2    1               eupenifeldin 2  9358 1431  7927 0.152917290
## 3    1 4-dehydroxy norpycnidione 5  9675  516  9159 0.053333333
## 4    1       4-hydroxyxenovulene B 4  9240 1937  7303 0.209632035
## 5    1                pycnidione 1  9370 1661  7709 0.177267876
## 6    1 4-hydroxy norxenovulene B 6  9611  695  8916 0.072312975
## 7    1                   untreated  9751   51  9700 0.005230233
## 8    2               xenovulene B 3  8951 1000  7951 0.111719361
## 9    2               eupenifeldin 2  9451 1557  7894 0.164744471
## 10   2 4-dehydroxy norpycnidione 5  8932  411  8521 0.046014330
## 11   2       4-hydroxyxenovulene B 4  9261  729  8532 0.078717201
## 12   2                pycnidione 1  9164 1694  7470 0.184853776
## 13   2 4-hydroxy norxenovulene B 6  9354  326  9028 0.034851400
## 14   2                   untreated  9382   33  9349 0.003517374
## 15   3               xenovulene B 3  9585  886  8699 0.092436098
## 16   3               eupenifeldin 2  9767 2313  7454 0.236817856
## 17   3 4-dehydroxy norpycnidione 5  9700  411  9289 0.042371134
## 18   3       4-hydroxyxenovulene B 4  9519  786  8733 0.082571699
## 19   3                pycnidione 1  9342 2442  6900 0.261400128
## 20   3 4-hydroxy norxenovulene B 6  9779  280  9499 0.028632785
## 21   3                   untreated  9772  136  9636 0.013917315
```

Statistical model

- Block design: One observation per compound in each replication
- Generalized linear model
- Binomial proportion for dead cells depend on Rep and Compound
- Logit-Link

```
fit <-  glm(cbind(Dead, Alive) ~ Rep + Compound,
            family=quasibinomial(link="logit"),
            data=toxy)

summary(fit)$dispersion
```

```
## [1] 129.9988
```

- Huge amound of overdispersion:
- The variance is almost 130 times higher than under simple binomial distribution

Deviance analysis

- Deviance analysis based on F-Test due to quasi-binomial assumption

```
anova(fit,
      test="F")

## Analysis of Deviance Table
##
## Model: quasibinomial, link: logit
##
## Response: cbind(Dead, Alive)
##
## Terms added sequentially (first to last)
##
##
##          Df Deviance Resid. Df Resid. Dev       F    Pr(>F)
## NULL                        20    13351.9
## Rep       2    178.2        18    13173.7  0.6855    0.5225
## Compound  6  11645.6        12     1528.1 14.9304 6.148e-05 ***
## ---
## Signif. codes:  0 '***' 0.001 '**' 0.01 '*' 0.05 '.' 0.1 ' ' 1
```

- The p-value for the variable compound is far smaller than $\alpha = 0.05$
- At least two compounds differ from each other with regard to their average proportion of dead cells

Estimated average proportions and their comparisons

Pairwise comparisons of average proportions $\hat{\pi}_j$ were run on logit scale. Hence, the tested null-hypothesis for each comparison was $H_0 : ln\left(\frac{\pi_j}{1-\pi_j}\right) - ln\left(\frac{\pi_{j'}}{1-\pi_{j'}}\right) = 0$. Back transformation to the response scale results in odds ratios $\frac{\hat{\pi}_j}{1-\hat{\pi}_j} / \frac{\hat{\pi}_{j'}}{1-\hat{\pi}_{j'}}$. Hence, back transformation from logit to response scale alters the H_0 such that $H_0 : \frac{\pi_j}{1-\pi_j} / \frac{\pi_{j'}}{1-\pi_{j'}} = 1$.

```
comp <- emmeans(fit,
                specs="Compound",
                contr="pairwise",
                type="response",
                adjust="mvt")
```

Estimated mean proportions of dead cells

```
##                        Compound        prob          SE  df   asymp.LCL   as
ymp.UCL
## 1                     untreated 0.007550266 0.005782888 Inf 0.00167346 0.0
3337510
## 2                  pycnidione 1 0.207168230 0.027687215 Inf 0.15809553 0.2
6664871
```

```
## 3             eupenifeldin 2 0.184836578 0.026188748 Inf 0.13888478 0.2
4172426
## 4              xenovulene B 3 0.100795816 0.020458394 Inf 0.06718123 0.1
4855122
## 5     4-hydroxyxenovulene B 4 0.122629081 0.022328644 Inf 0.08513631 0.1
7350178
## 6 4-dehydroxy norpycnidione 5 0.046832482 0.014268929 Inf 0.02558786 0.0
8419170
## 7 4-hydroxy norxenovulene B 6 0.044942643 0.013896502 Inf 0.02434141 0.0
8152277
```

A graphical overview about this table is given in the manuscript in figure 5 a.

Mean comparisons (contrast tests)

```
##                                                         contrast ratio p
.value
## 1                                       untreated / pycnidione 1 0.029
<0.001
## 2                                     untreated / eupenifeldin 2 0.034
<0.001
## 3                                     untreated / xenovulene B 3 0.068
 0.012
## 4                          untreated / (4-hydroxyxenovulene B 4) 0.054
 0.004
## 5                      untreated / (4-dehydroxy norpycnidione 5) 0.155
 0.252
## 6                      untreated / (4-hydroxy norxenovulene B 6) 0.162
 0.281
## 7                                pycnidione 1 / eupenifeldin 2 1.152
 0.997
## 8                                pycnidione 1 / xenovulene B 3 2.331
 0.036
## 9                     pycnidione 1 / (4-hydroxyxenovulene B 4) 1.870
 0.202
## 10                pycnidione 1 / (4-dehydroxy norpycnidione 5) 5.318
<0.001
## 11                pycnidione 1 / (4-hydroxy norxenovulene B 6) 5.553
<0.001
## 12                             eupenifeldin 2 / xenovulene B 3 2.023
  0.15
## 13                  eupenifeldin 2 / (4-hydroxyxenovulene B 4) 1.622
 0.525
## 14              eupenifeldin 2 / (4-dehydroxy norpycnidione 5) 4.615
<0.001
## 15              eupenifeldin 2 / (4-hydroxy norxenovulene B 6) 4.819
<0.001
## 16                  xenovulene B 3 / (4-hydroxyxenovulene B 4) 0.802
  0.99
## 17              xenovulene B 3 / (4-dehydroxy norpycnidione 5) 2.281
 0.319
## 18              xenovulene B 3 / (4-hydroxy norxenovulene B 6) 2.382
 0.269
## 19   (4-hydroxyxenovulene B 4) / (4-dehydroxy norpycnidione 5) 2.845
 0.076
## 20   (4-hydroxyxenovulene B 4) / (4-hydroxy norxenovulene B 6) 2.970
```

```
 0.061
## 21 (4-dehydroxy norpycnidione 5) / (4-hydroxy norxenovulene B 6) 1.044
>0.999
```

A graphical overview about this table is given in the manuscript in figure 5 b.
